# IL-17RA-Signaling Modulates CD8+ T Cell Survival and Exhaustion During *Trypanosoma cruzi* Infection

**DOI:** 10.3389/fimmu.2018.02347

**Published:** 2018-10-11

**Authors:** Jimena Tosello Boari, Cintia L. Araujo Furlan, Facundo Fiocca Vernengo, Constanza Rodriguez, María C. Ramello, María C. Amezcua Vesely, Melisa Gorosito Serrán, Nicolás G. Nuñez, Wilfrid Richer, Eliane Piaggio, Carolina L. Montes, Adriana Gruppi, Eva V. Acosta Rodríguez

**Affiliations:** ^1^Departamento de Bioquímica Clínica, Facultad de Ciencias Químicas, Universidad Nacional de Córdoba, Córdoba, Argentina; ^2^Centro de Investigaciones en Bioquímica Clínica e Inmunología, CONICET, Córdoba, Argentina; ^3^SiRIC TransImm “Translational Immunotherapy Team,” Translational Research Department, Research Center, PSL Research University, INSERM U932, Institut Curie, Paris, France; ^4^Centre d'Investigation Clinique Biothérapie CICBT 1428, Institut Curie, Paris, France

**Keywords:** IL-17, chagas disease, immunity, cellular, CD8+ T cells, exhausted T cells, effector function, apoptosis

## Abstract

The IL-17 family contributes to host defense against many intracellular pathogens by mechanisms that are not fully understood. CD8+ T lymphocytes are key elements against intracellular microbes, and their survival and ability to mount cytotoxic responses are orchestrated by several cytokines. Here, we demonstrated that IL-17RA-signaling cytokines sustain pathogen-specific CD8+ T cell immunity. The absence of IL-17RA and IL-17A/F during *Trypanosoma cruzi* infection resulted in increased tissue parasitism and reduced frequency of parasite-specific CD8+ T cells. Impaired IL-17RA-signaling *in vivo* increased apoptosis of parasite-specific CD8+ T cells, while *in vitro* recombinant IL-17 down-regulated the pro-apoptotic protein BAD and promoted the survival of activated CD8+ T cells. Phenotypic, functional, and transcriptomic profiling showed that *T. cruzi*-specific CD8+ T cells derived from IL-17RA-deficient mice presented features of cell dysfunction. PD-L1 blockade partially restored the magnitude of CD8+ T cell responses and parasite control in these mice. Adoptive transfer experiments established that IL-17RA-signaling is intrinsically required for the proper maintenance of functional effector CD8+ T cells. Altogether, our results identify IL-17RA and IL-17A as critical factors for sustaining CD8+ T cell immunity to *T. cruzi*.

## Introduction

IL-17A and IL-17F were initially associated with the pathogenesis of autoinflammatory and autoimmune disorders (1). Nonetheless, the primary function of these cytokines is likely host protection against microbes. Indeed, mice deficient in IL-17A and/or IL-17F are highly susceptible to infection with a wide array of fungi and extracellular bacteria, as well as viruses and parasites (2, 3). We previously demonstrated that the IL-17 family of cytokines plays a critical role in host survival by regulating exuberant inflammation and immunopathology during infection with the protozoan *Trypanosoma cruzi* (4, 5). Additional data from our laboratory suggests that IL-17 plays additional protective roles against *T. cruzi* by modulating adaptive immunity.

The IL-17 family is comprised of 6 members, IL-17A to IL-17F, which have different cellular sources and expression patterns but often show overlapping activities. The cytokines signal through a receptor complex composed of at least two identical or different subunits of the IL-17R family (IL-17RA to IL-17RE). IL-17RA is the common signaling subunit used by at least four ligands: IL-17A, IL-17C, IL-17E, and IL-17F. IL-17 members regulate inflammation by recruiting and activating neutrophils, NK cells, and other cells of the innate immune system and by inducing several pro-inflammatory mediators (i.e., cytokines, chemokines, microbial peptides, and metalloproteinases). In addition, these cytokines play important roles during adaptive immune responses, including the modulation of germinal center reactions, as well as the regulation of Th1 and CD8+ cellular responses (6–9).

Infection with *T. cruzi* is known to cause Chagas disease, the third most frequent parasitic disease worldwide that is endemic in Latin America and is increasing globally due to migratory flows. Disease progression, from asymptomatic to severe cardiac and digestive forms, is linked to parasite heterogeneity and variable host immune responses. CD8+ T cell mediated immunity is essential for parasite control throughout the various stages of infection, although it is not sufficient for complete parasite elimination (10, 11). CD8+ T cell deficient mice are extremely susceptible to infection (12, 13), and strategies that improve specific CD8+ T cell responses result in increased host protection (11, 14, 15). This finding, coupled with the fact that parasite persistence correlates with disease severity in human and experimental models of *T. cruzi* infection (16, 17), have prompted efforts to better understand the mechanisms that promote anti-parasitic CD8+ T cell immunity.

The general features of protective CD8+ T cell responses (as defined with model viral and bacterial infections) consist of the generation and expansion of short-lived, highly functional effector populations with antimicrobial activities. After the pathogen has been cleared, most of the effector cells die and the few remaining cells differentiate into memory T cells, contributing to long-lived immunological protection (18). Persistently activated CD8+ T cells can become dysfunctional during certain chronic infections, which results in a hierarchical loss of effector functions and proliferation potential, as well as sustained expression of multiple inhibitory receptors. Severe exhaustion leads to pathogen-specific cells that are prone to deletion and have reduced ability to produce cytokines and degranulate. These exhausted CD8+ T cells fail to provide optimal protection and contribute to poor pathogen control (19). In addition to antigen-specific and co-stimulatory signals, which are the driving forces behind CD8+ T cell responses, various cytokines (i.e., IL-21, IL-2, IL-12) are required to provide the “third-signal” essential for protective CD8+ T cell immunity (20, 21).

Herein, we demonstrate that IL-17RA signaling is required for the maintenance of robust, specific CD8+ T cell responses that contribute to host resistance to *T. cruzi*. We show that absence of IL-17RA signaling during *T. cruzi* infection alters the transcriptional program of effector CD8+ T cells, and consequently affects their survival, effector function, and exhaustion. Our findings are relevant for gaining a better understanding of the role of the IL-17 family in the orchestration of protective immunity against infections.

## Materials and methods

### Mice

Mice used for experiments were sex- and age-matched (6 to 10 weeks-old). C57BL/6 mice were obtained from School of Veterinary, La Plata National University (La Plata, Argentina). IL-17RA KO mice were provided by Amgen Inc. (Master Agreement N° 200716544-002). IL-17A/IL-17F double KO mice (22) were kindly provided by Dr Immo Prinz. CD45.1 C57BL/6 mice (B6.SJL-*Ptprca Pepcb*/Boy) and CD8α KO mice (B6.129S2-Cd8atm1Mak/J) were obtained from The Jackson Laboratories (USA). CD45.1 × CD45.2 F1 WT mice bred in our animal facility. All animals were housed in the Facultad de Ciencias Químicas, Universidad Nacional de Córdoba.

### Parasites and experimental infection

Bloodstream trypomastigotes of the Tulahuén strain of *T. cruzi* were obtained from BALB/c mice infected 10 days earlier. For experimental infection mice were inoculated intraperitoneally with 0.2 ml PBS containing 5 × 10^3^ trypomastigotes (usual dose) or doses of 500 and 5 × 10^4^ trypomastigotes when indicated. For infection of transferred CD8α knockout mice, parasite dose was 1,000 trypomastigotes.

### Quantification of parasite DNA in tissues

Genomic DNA was purified from 50 μg of tissue (spleen, liver, and heart) using TRIzol Reagent (Life Technologies) following manufacturer's instructions. Satellite DNA from *T. cruzi* (GenBank AY520036) was quantified by real time PCR using specific Custom Taqman Gene Expression Assay (Applied Biosystem) using the primer and probe sequences described by Piron et al. (23). A sample was considered positive for the *T. cruzi* target when CT < 45. Abundance of satellite DNA from *T. cruzi* was normalized to GAPDH abundance (Taqman Rodent GAPDH Control Reagent, Applied Biosystem) and expressed as arbitrary units.

### Cells and culture

CD8+ T cells were isolated from the spleen by magnetic negative selection or cell sorting from pools of at least 3-5 mice. For magnetic cell purification the EasySep™ Mouse CD8+ T Cell Isolation Kit (Stemcell Technologies) were used according to the manufacturer's protocol. For cell sorting, spleen cell suspensions were surface stained and CD3+CD8+ T cells were sorted with a FACSAria II (BD Biosciences). During *in vitro* studies, CD8+ T cells (2 × 10^5^) purified by magnetic selection were stimulated in 96-well plates coated with anti-CD3/anti-CD28 Abs (eBioscience, 2 and 1 μg/ml respectively) and incubated during 24 h in the presence of 100 ng/ml of recombinant IL-17A, IL-17F, IL-17C, and IL-17E (ImmunoTools, GmbH) and/or 50 ng/mL of IL-21 and TNF (ImmunoTools, GmbH). For camptothecin challenge, the drug was added in a concentration of 5 μM from the beginning of the culture.

### Antibodies and flow cytometry

Cell suspensions were washed in PBS and incubated with LIVE/DEAD Fixable Cell Dead Stain (eBioscience) during 15 min at RT. Next, cells were washed and incubated with fluorochrome labeled-Abs for 20 min at 4°C (see below for details). To detect *T. cruzi* specific CD8+ T cells, H-2K(b) *T. cruzi* trans-sialidase amino acids 567-574 ANYKFTLV (TSKB20) APC-Labeled Tetramer (NIH Tetramer Core Facility) were incubated 20 min at 4°C before further surface staining with additional Abs. After further surface staining, cells were washed and acquired in a FACSCanto II (BD Biosciences). Blood was directly incubated with the indicated antibodies and erythrocytes were lysed with a 0.87% NH_4_Cl buffer previously to acquisition. For Ki-67 intranuclear staining, cells were first stained on surface, washed and then fixed, permeabilized and stained with Foxp3/Transcription Factor Staining Buffers (eBioscience) following eBioscience One-step protocol: intracellular (nuclear) proteins.

Flow cytometry and/or cell sorting was performed with a combination of the following Abs (BD Biosciences, Biolegend, eBioscience, Life Technologies, Santa Cruz Biotechnology or Cell Signaling): FITC/AF488-labeled anti-mouse: CD8 (53-6.7), anti-CD25 (7D4), CD69 (H1.2F3), CD44 (IM7), Bcl-2 (10C4), IgG (polyclonal goat), and anti-rat IgG (polyclonal goat); PE-labeled anti-mouse: Fas (Jo2), CTLA-4 (UC10-4B9), PD-1 (RMP1-30), Ki-67 (SolA15), IL-17RA (PAJ17R), CD120b (TR75-89), Bim (rabbit. C34C5), rabbit IgG control isotype (DA1E); PECy7-labeled anti-mouse: CD8 (53-6.7), PD-1 (RMP1-30), FAS (Jo2) and anti-CD25 (P61.5); PerCPCy5.5-labeled anti-mouse: CD3 (145-2C11), CD45.2 (104); PerCP-eFluor 710 labeled anti-mouse: TIGIT (GIGD7); APC-Cy7/AF780-labeled anti-mouse: CD45.1 (A20) and CD62L (MEL-14); Biotin-labeled anti-mouse: BTLA (8F4) and PE-labeled Streptavidin; unlabeled anti-mouse: Bcl-xL (rabbit, 54H7), Bcl-2-associated death promoter (BAD) (rabbit, C-20), Bax (rabbit, P-19).

### Quantification of IL-21

The concentration of IL-21 in the plasma of in plasma of infected WT and IL-17RA KO mice was assessed by ELISA using paired specific Abs (eBiosciences) according to standard protocols.

### Evaluation of proliferation and apoptosis

*T. cruzi*-infected WT and IL-17RA KO mice were given BrdU (1 mg/ml, Sigma-Aldrich)/1% sucrose in the drinking water (carefully protected from light) ad libitum. After 5 days, mice were sacrificed and spleen mononuclear cells were collected and stained with surface antibodies. Incorporated BrdU was detected with a BrdU Flow kit according to the manufacturer's specifications (BD Biosciences).

Apoptosis was determined by Annexin V and 7AAD staining according to the manufacturer's specifications (BD Biosciences). Mitochondrial depolarization (ψ) was measured by FACS using 50 nM TMRE (Invitrogen) as described (24). When indicated, 7AAD or LIVE/DEAD Fixable Aqua dead cell stain was used in combination to identify live, early apoptotic and late apoptotic/necrotic cells.

### Determination of CD8+ T cell effector function *in vitro*

Spleen cell suspensions were cultured during 5 h with medium, 5 μg/ml TSKB20 (ANYKFTLV) peptide (Genscript Inc.), 50 nM PMA plus 0.5 μg/ml ionomycin (Sigma-Aldrich) in the presence of Monensin and Brefeldin A (eBioscience). When indicated a PE-labeled anti-CD107a mAb (eBioscience, eBio1D4B) was included during the culture period. After culture, the cells were surface stained, fixed and permeabilized with BD Cytofix/Cytoperm and Perm/Wash (BD Biosciences) according manufacturer's instruction. Cells were incubated with FITC-labeled antibodies to IFNγ (eBioscience, XMG1.2) or Perforin-1 (eBoscience, eBioOMAK-D), PerCP/PerCP-eFluor 710 labeled antibody to TNF (Biolegend, MP6-XT22) or Granzyme A (eBiosciences, GzA-3G8.5) and/or APC/AF647-labeled antibody to TNF (Biolegend, MP6-XT22) or Granzyme B (Biolegend, GB11). Stained cells were acquired on FACSCanto II (BD Biosciences).

### Adoptive cell transfer

Competitive adoptive transfer experiments were performed by i.v. injection of recipient CD45.1+/CD45.2+ WT mice (F1 hybrid obtained by crossing CD45.1+ and CD45.2+ WT mice) or CD8α knockout mice with a mixture 1:1 of CD8+ T cells purified from spleen of CD45.1+ WT mice and CD45.2+ IL-17RA KO mice. Total 5 × 10^6^ cells and 15 × 10^6^ cells were injected in WT and CD8α knockout recipients, respectively. Recipient mice were immediately infected and frequency of the injected CD45.1+ WT and CD45.2+ IL-17RA KO cells within the total T cell population were determined in blood, spleen and liver at different days pi.

To address the relevance of CD8+ T cell-intrinsic IL-17RA signaling for *T. cruzi* infection progression, CD8α knockout hosts were injected i.v. with the same number (7 × 10^6^) of CD8+ T cells purified from spleen of WT or IL-17RA KO mice and immediately infected. Transfer of equal number of CD8+ T cells in both mouse groups was evaluated 1 day after injection by analyzing the frequency of CD8+ T cells in blood. The progression of the infection was followed by the quantification of parasitemia and survival. Infected non-transferred CD8α knockout mice were used as control.

### Treatment with neutralizing anti- IL-17A and PD-L1 Abs

To block the PD-1/PD-L1 pathway, infected WT and IL-17RA KO mice were injected i.p. with 200 μg rat anti-mouse PD-L1 antibody (10F.9G2, BioXcell) or total rat IgG isotype control (Jackson Research) every 3 days from 15 dpi until sacrifice at day 21 dpi. Infection matched WT mice were assayed in parallel as controls.

For the *in vivo* neutralization of IL-17A, infected WT mice were injected i.p. with 200 μg rat anti-mouse IL-17 antibody (17F3, BioXcell) or rat total IgG1 isotype control (MOPC-21, BioXcell) every 3 days from day 13 dpi until sacrifice at 21 dpi. Infection-matched IL-17RA KO mice were assayed in parallel as controls.

### Real-time quantitative PCR and DNA microarray

RNA from total spleen tissue was purified using TRIzol Reagent (Life Technologies) following manufacturer's instructions. Oligo-dT and a MMLV reverse transcriptase kit (Invitrogen) were used for cDNA synthesis. IL-17RA, IL-17RC, and IL-17RD transcripts were quantified by real-time quantitative PCR on an ABI PRISM 7700 Sequence Detector (Perkin-Elmer Applied Biosystems) with Applied Biosystems predesigned TaqMan Gene Expression Assays and reagents according to the manufacturer's instructions. The following probes were used: Mm00434214_m1 for IL-17RA, Mm00506606_m1 for IL-17RC and Mm00460340_m1 for IL-17RD. For each sample, the mRNA abundance was normalized to the amount of 18S rRNA (Mm03928990_g1) and is expressed as arbitrary units (AU).

For gene-expression analysis, CD8+ T cells were purified by FACS from the spleen of non-infected and 22-day infected WT and IL-17RA KO mice and lysed with TRIzol reagent. Total RNA was extracted with the RNAeasy Mini Kit (Qiagen). RNA quality was verified in an Agilent Bioanalyzer and measured with a Nanodrop 1000 (Thermo Scientific). cDNA was hybridized on Affymetrix Mouse Gene 2.1 ST arrays as described elsewhere.

### Analysis of microarray data

The microarray data from this publication have been deposited to the GEO database (https://www.ncbi.nlm.nih.gov/geo/) and assigned the identifier: GSE104886. Gene expression data was normalized using RMA algorithm on custom Brainarray CDF (v.22.0.0 ENTREZG). Plots of differential expressed genes in spleen CD8 T cells at day 0 post infection and day 22 post infection in WT and IL-17RA KO samples were defined using fold change [|log2FC| >log2(1.2)]. Bioinformatic analyses were performed with R software environment. Heatmaps were produced using Heatmapper (http://www.heatmapper.ca) and show gene expression of CD8+ T cells from WT and IL-17RA KO normalized by the average of the gene expression in non-infected counterparts, except specified. Gene Set Enrichment Analyses (GSEA) between the different spleen CD8 T cell samples were done using specific gene sets from the Molecular Signatures DB (MSigDB). Genes induced by the infection in WT and IL-17RA KO mice but showing significantly higher expression in IL-17RA KO mice were uploaded to Ingenuity Pahtway Analysis (Ingenuity® Systems, www.ingenuity.com) for the analysis of “Disease and bio-function,” “Canonical pathway,” and “Up-stream regulators.” It was considered significantly activated (or inhibited) with an overlap *p* ≤ 0.05 and an IPA activation Z-score as defined under each specific analysis category on the IPA website.

### Statistics

Statistical significance of comparisons of mean values was assessed as indicated by a two-tailed Student's *t* test, two way ANOVA followed by Bonferroni's posttest and Gehan-Breslow-Wilcoxon Test using GraphPad software. *P* < 0.05 was considered statistically significant.

## Results

### IL-17RA and IL-17A/IL-17F deficiencies compromise parasite control and reduce the magnitude of the specific CD8+ T cell response during *T. cruzi* infection

**Figure 1:**
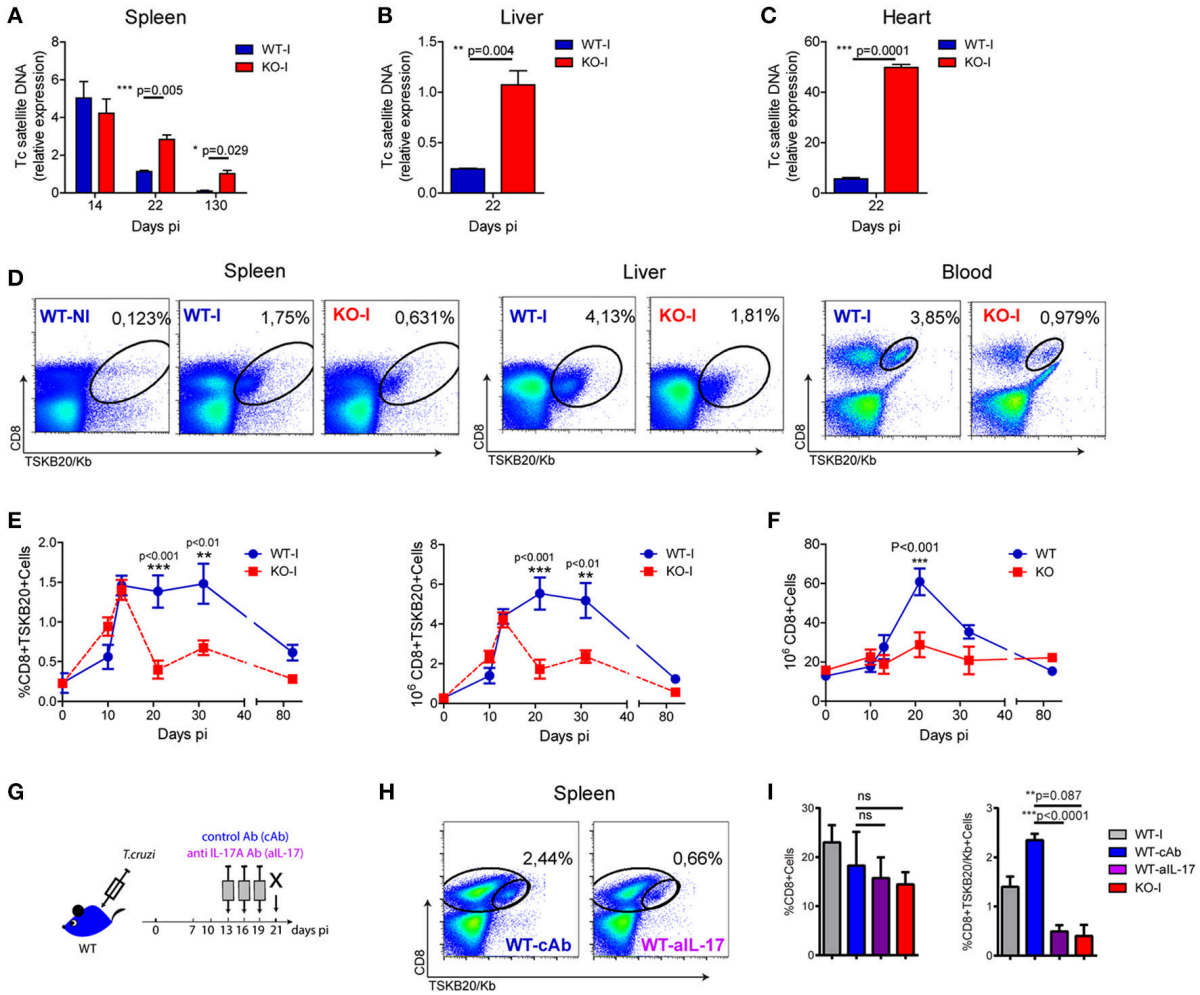
Absence of IL-17RA signaling results in increased tissue parasitism and a reduced magnitude of parasite-specific CD8+ T cell responses. **(A-C)** Relative amount of *T. cruzi* satellite DNA in spleen **(A)**, liver **(B)** and heart **(C)** of infected WT and IL-17RA KO (KO) mice determined at the indicated dpi. Murine GAPDH was used for normalization. Data are presented as mean ± SD, *N* = 5 mice. *P* values calculated with two-tailed *T* test. **(D)** Representative plots of CD8 and TSKB20/Kb staining in spleen, liver and blood of WT and KO mice at 22 dpi (WT-I and KO-I, respectively). A representative plot of the staining of splenocytes from non-infected WT mice (WT-N) is shown for comparison. **(E)** Percentage and cell numbers of TSKB20/Kb+ CD8+ T cells and **(F)** cell numbers of total CD8+ T cells determined in spleen of WT and IL-17RA KO mice at different dpi. Data shown as mean ± SD, *N* = 5−8 mice. *P* values calculated using two-way ANOVA followed by Bonferroni‘s post-test. **(G)** Experimental layout of IL-17 neutralization in infected WT mice injected with control isotype or anti-IL-17 Abs (WT-cAb and WT-aIL-17, respectively). **(H)** Representative plots of CD8 and TSKB20/Kb staining in spleen and liver infected control and treated WT mice. **(I)** Percentage of total and TSKB20/Kb+ CD8+ T cells infected control and treated WT mice. Results from infection-matched WT (WT-I) and KO mice (KO-I) are shown for comparison. Data are representative of five **(A–F)**, and two **(G–I)** independent experiments.

We previously showed that IL-17RA-signaling and IL-17-secreting B cells are required for host survival during *T. cruzi* infection by, at least in part, regulating innate immunity and inflammation (4, 5). In addition, histological data from these initial studies suggested that infected IL-17RA knockout (KO) mice exhibited increased parasite levels in tissues, but similar levels of parasitemia, compared with those of their infected wildtype (WT) counterparts (4). To confirm this and to study possible additional protective mechanisms mediated by IL-17, we evaluated tissue parasitism in infected WT and IL-17RA KO mice. As determined by the amounts of parasite DNA quantified in the spleen, we observed that *T. cruzi* infected IL-17RA KO mice controlled tissue parasitism similar to WT mice during the first 2 weeks of infection (Figure 1A). However, IL-17RA KO mice failed to reduce the parasite burden at later stages of infection, as attested by the increased parasite load in the spleen at 22 and 130 days post-infection (dpi) (Figure 1A). Furthermore, increased parasite levels were also detected in other organs, such as liver and heart (Figures 1B,C).

Given that the aforementioned result suggested a deteriorated adaptive immune response, we focused on studying the role of IL-17RA in the development of parasite-specific CD8+ T cells. To this end, we examined the generation of CD8+ T cells specific for the immunodominant epitope TSKB20 (*T. cruzi* trans-sialidase amino acids 569-576 –ANYKFTLV–) (25) in infected IL-17RA KO and WT mice. We determined that the frequency of TSKB20-specific CD8+ T cells was reduced in the spleen, liver, and blood of infected IL-17RA KO mice at 20 dpi (Figure 1D). Kinetics studies showed that the frequency and absolute numbers of TSKB20-specific CD8+ T cells in the spleen of infected IL-17RA KO mice was similar to that of infected WT control mice up to 14 dpi, but these values dropped dramatically soon after (Figure 1E). Remarkably, total CD8+ T cell numbers at 20 dpi were also reduced in the spleen of infected IL-17RA KO mice (Figure 1F). In agreement with the notion that IL-17A and IL-17F are the major IL-17RA-signaling cytokines that modulate immune responses, IL-17A/IL-17F double knockout (DKO) mice also showed increased tissue parasitism in spleen and liver (Supplementary Figure 1A) and reduced frequency of parasite-specific CD8+ T cells in comparison to WT control mice (Supplementary Figure 1B).

Considering the kinetics of the TSKB20-specific CD8+ T cell immunity observed in infected IL-17RA KO mice (Figure 1E), along with our results showing that IL-17 production peaked around 14 dpi (4, 5), we speculated that IL-17RA signaling plays a role during the expansion and maintenance of parasite-specific CD8+ T cells after priming. To test this, we performed neutralization experiments in which infected WT mice were injected with anti-IL-17A antibodies (Abs) from 13 to 19 dpi (Figure 1G). A blockade of IL-17 signaling, specifically during the expansion/maintenance phase, resulted in a significant decrease in the frequency of TSKB20-specific T cells (Figure 1H). Indeed, mice treated with anti-IL-17 once the CD8+ T cell response was already established (from 13 to 19 dpi), showed a frequency of parasite-specific T cells comparable to that observed in infected IL-17RA KO mice that were devoid of IL-17 signaling throughout the entire infection period (Figure 1I).

### Absence of IL-17RA signaling during *T. cruzi* infection reduces the survival of total and specific CD8+ T cells

**Figure 2:**
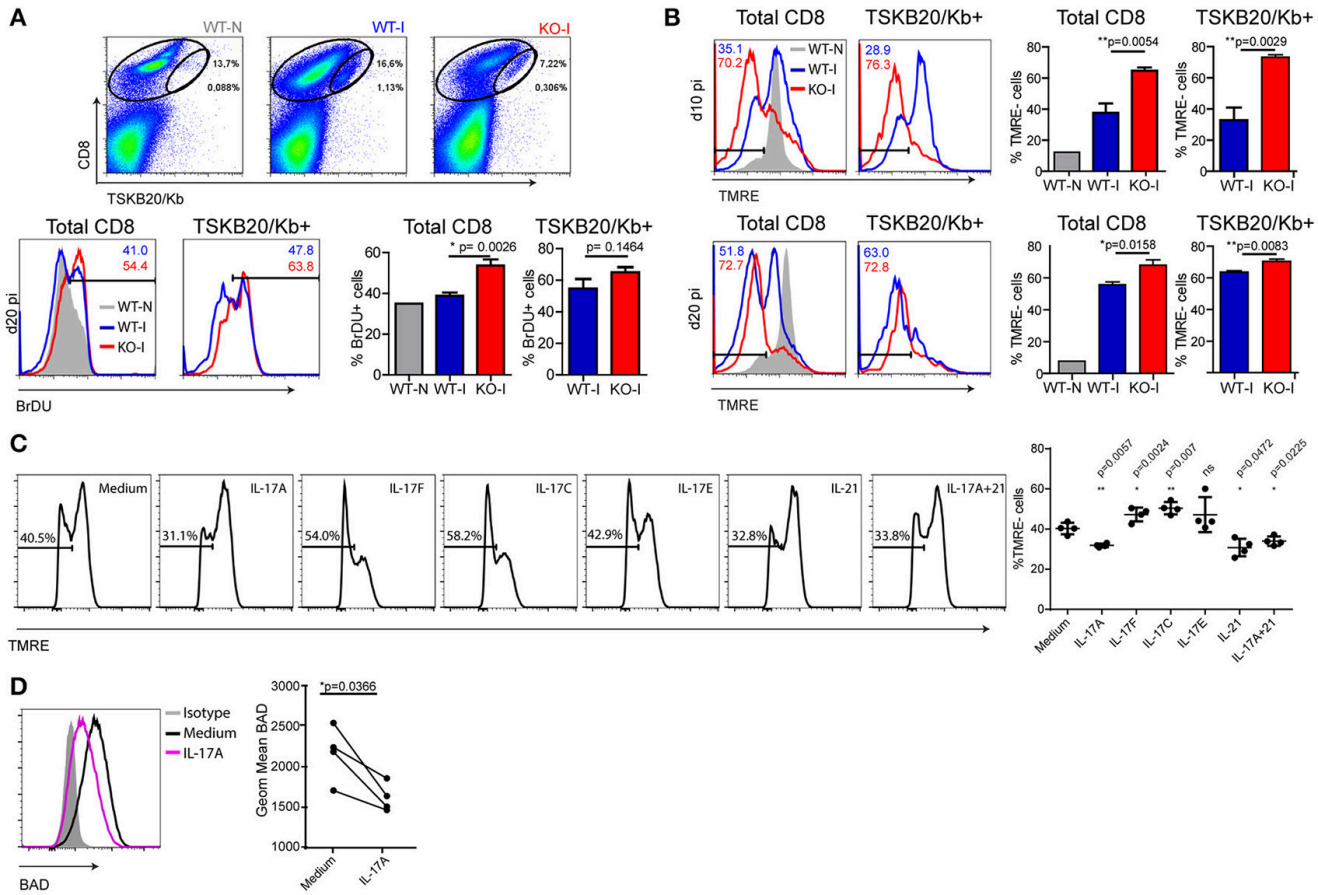
IL-17RA signaling during *T. cruzi* infection promotes survival of CD8+ T cells. **(A)** Representative dot plots of CD8 and TSKB20/Kb staining and representative histograms of BrDU in cell suspensions from the spleen of WT and KO mice at 0 dpi (WT-N) and 20 dpi (WT-I and KO-I) and within the indicated total and TSKB20/Kb+ CD8+ T cell gates. Numbers in the histograms indicate the frequency of BrDU+ cells from the corresponding colored group. Histograms are representative of one out of five mice. Bars in the statistical analysis represent data as mean ± SD, *N* = 5 mice. *P* values were calculated with two-tailed *T* test. **(B)** Representative histograms of TMRE staining in total (left) and TSKB20/Kb+ (right) CD8+ T cells from the spleen of WT and IL17RA KO mice at 10 dpi (top) and 20 dpi (bottom). Numbers indicate the frequency of TMRE- (apoptotic) cells in total and TSKB20/Kb+ CD8+ T cells from the corresponding colored group. Gray tinted histogram show staining in CD8+ T cells from non-infected WT mice (WT-N). Histograms are representative of one out of five mice. Bars in the statistical analysis represent data as mean ± SD, *N* = 5 mice. *P* values were calculated with two-tailed *T* test. **(C, D)** Representative histograms of TMRE **(C)** and BAD **(D)** stainings in cultures of purified CD8+ T cells activated with coated anti-CD3 and anti-CD28 in the presence of the indicated cytokines. Statistical analysis in **(C)** shows the frequency of TMRE- (apoptotic) cells in each biological replicate (*N* = 4 mice) and the mean ± *SD*. *P* values calculated with paired two-tailed *T* test (ns, not significant). Statistical analysis in **(D)** represent protein expression as the geometric mean of fluorescence intensity in each replicate (*N* = 4 mice). Lines link paired samples. *P* values calculated with paired two-tailed *T* test. Data in **(A-D)** are representative of three and two independent experiments, respectively.

Considering the conventional kinetics of T cell responses (18), it was conceivable that the abortive CD8+ T cell response observed in infected IL-17RA KO mice could be a consequence of reduced cell expansion and/or increased contraction. To address the role of IL-17RA in CD8+ T cell proliferation, we assessed the *in vivo* incorporation of BrdU between 15 and 20 dpi, as these time points directly preceded the dramatic decrease in the numbers of specific CD8+ T cells in infected IL-17RA KO mice. The results depicted in Figure 2A demonstrate that the absence of IL-17RA does not compromise proliferation of CD8+ T cells during *T. cruzi* infection. Complementary studies of e*x vivo* detection of the Ki-67 antigen led to similar conclusions (Supplementary Figure 2A). We then tested a possible role of IL-17RA signaling in supporting CD8+ T cell survival. In comparison to their infected WT counterparts, total and TSKB20-specific CD8+ T cells from infected IL-17RA KO mice showed significantly higher frequencies of apoptotic cells as early as 10 dpi and also at later time points (20 dpi) (Figure 2B). Similar results were obtained by analyzing Annexin V+/7AAD- cells (Supplementary Figure 2B). In the same direction, total and parasite-specific CD8+ T cells from infected WT mice treated with anti-IL-17A Abs (Figure 1H) showed higher percentage of apoptotic cells than their counterparts from WT mice, and exhibited levels of apoptosis similar to that of infected IL-17RA KO mice (Supplementary Figure 2C).

Given the results described above, we evaluated whether cytokines that signal through IL-17RA were able to directly increase survival of activated CD8+ T cells. CD8+ T cells purified from the spleen of non-infected WT mice were activated for 24 h with plastic-coated anti-CD3 and anti-CD28 Abs in the presence of recombinant cytokines. The addition of IL-17A, but not IL-17F, IL-17C, or IL-17E, lead to a modest but significant reduction in the percentage of apoptotic activated CD8+ T cells (Figure 2C). Of note, the direct anti-apoptotic effect of IL-17 in CD8+ T cells was comparable to that of IL-21, a well-recognized survival factor for this population (26). We also determined that CD8+ T cells purified from the spleen of IL-17RA KO mice and activated *in vitro* were more susceptible to cell death (apoptosis and necrosis) induced by camptothecin (CPT) challenge than CD8+ T cells from WT mice (Supplementary Figure 2D). As expected based on the lack of IL-17RA expression, IL-17 was not able to reduce activation- or CPT-induced cell death in CD8+ T cells from IL-17RA KO mice. However, IL-17 also failed to reduce the cell death triggered by CPT in CD8+ T cells from WT mice. The differential effect of IL-17 in protecting CD8+ T cells from TCR- but not CPT-induced cell death may result from differences in the apoptotic signaling pathways triggered by these two treatments (27, 28). In an attempt to elucidate the mechanisms underlying the pro-survival effects of IL-17A against activation-induced apoptosis, we evaluated the expression of pro- and anti-apoptotic members of the Bcl-2 superfamily that are critically involved in the regulation of T cell death (29). We determined that IL-17A significantly downregulated the expression of Bad (Figure 2D). Little to no changes were observed in other members such as Bcl-xL, Bcl-2, Bim, and Bax (Supplementary Figure 2E). Altogether, these results indicate that IL-17A may promote CD8+ T cell survival by down-regulating pro-apoptotic factors in the Bcl-2 family.

### Absence of IL-17RA perturbs the transcriptional program of CD8+ T cells activated during *T. cruzi* infection

**Figure 3:**
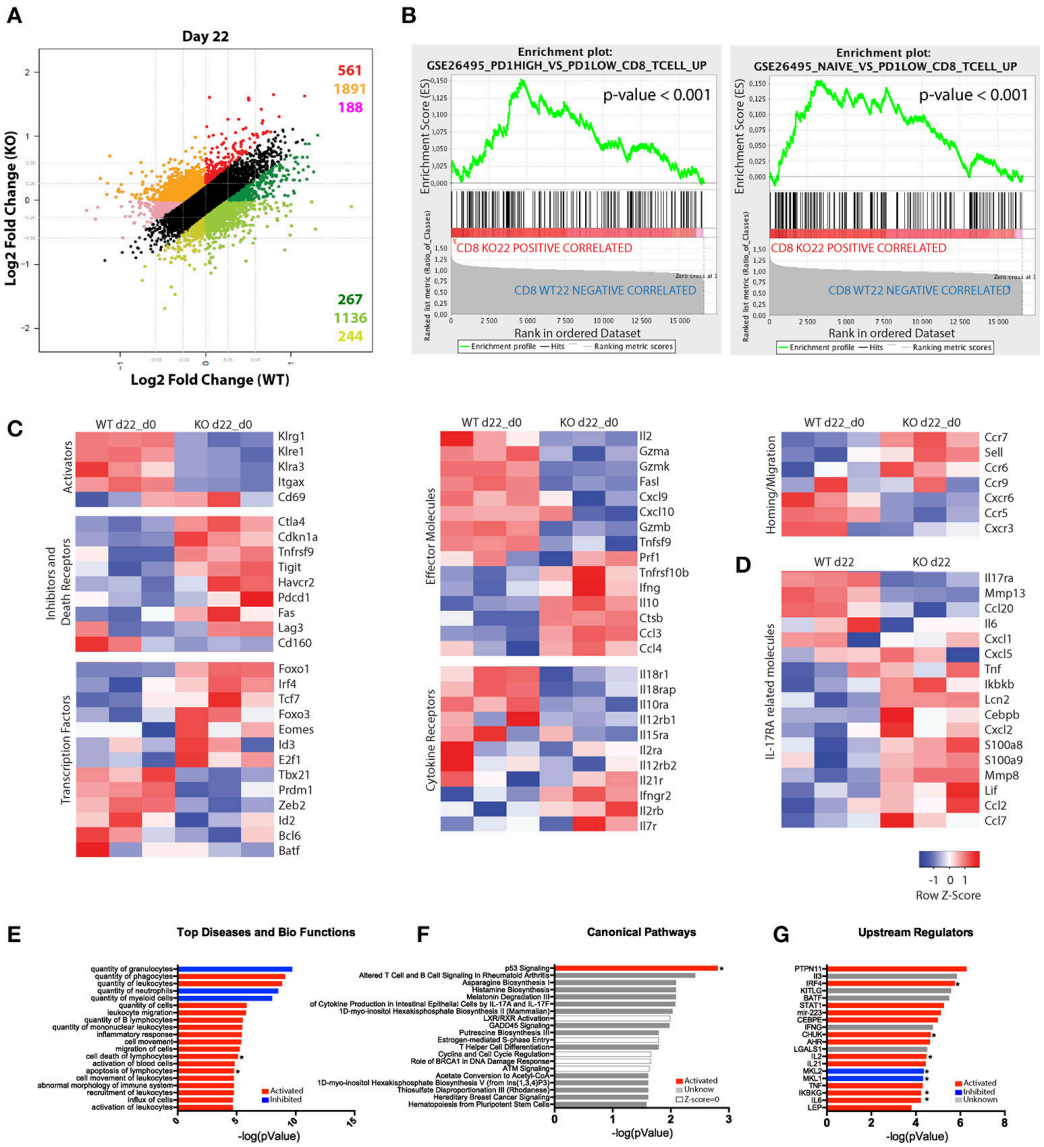
Substantial differences in the gene-expression profile of CD8+ T cells from *T. cruzi*-infected IL-17RA KO and WT mice. **(A–F)** Microarray analysis of purified CD8+ T cells from infected WT (WT-I) and IL-17RA KO (KO-I) mice (22 dpi) and non-infected controls (WT-N and KO-N). *N* = 3 mice per group. **(A)** Dot plot displaying the number of genes that show a significant ≥1.2-fold difference in fold change expression of WT-I and KO-I relative to non-infected counterparts. Colors indicate sets of genes with different expression patterns. **(B)** Top two enrichment plots in KO-I (*p* < 0.001) determined by supervised analysis of all infection induced genes in WT-I and KO-I using GSEA26495 and GSEA41867 (MSigDB C5BP) signature gene sets. **(C)** Heat maps of expression of selected genes according to categories relevant to CD8+ T cell biology. The gene expression values of each sample were normalized by division to the average values of the corresponding samples from non-infected counterparts. **(D)** Heat map of expression of genes encoding molecules associated to IL-17RA signaling. Gene expression values represent non-normalized data from 3 mice. **(E–G)** IPA of genes induced by the infection but showing significantly higher expression in IL-17RA KO mice (red genes in **A**). Top 20 “Diseases and Bio-functions” **(E)**, “Canonical pathways” **(F)**, and “Up-stream regulators” **(G)** that were most significantly altered (*P* < 0.05 with Fischer's exact test) in KO mice are shown. The activation Z-score was calculated to predict activation (red), inhibition (blue), categories where no predictions can be made and unknown results (with Z-score close to 0). Categories significantly activated or inhibited according to Z-score are marked with a star.

To gain further insights into the role of IL-17RA signaling during *T. cruzi* infection, we determined the gene expression profiles of purified CD8+ T cells using Affymetrix microarrays. Using gene expression data normalized to the corresponding non-infected samples, we compared the transcriptional changes induced by infection in CD8+ T cells from IL-17RA KO mice vs. WT mice in a fold change/fold change plot. We identified 4287 genes that showed significant expression differences (greater than 1.2 fold; *p* < 0.05) and grouped these genes into different sets according to the differential expression patterns (Figure 3A). Remarkably, 1,647 genes (pink, orange and red sets) were expressed at higher level in CD8+ T cells from IL-17RA KO infected mice in comparison to WT counterparts, whereas 2,640 genes (light green, green and dark sets) were expressed at lower levels. To further evaluate the biological characteristics of the transcriptional landscape of CD8+ T cells from infected IL-17RA KO vs. WT mice, we performed gene-enrichment set analysis (GSEA) for the genes that were differentially expressed. After conducting a broad analysis using gene ontology, as well as immunological and curated databases, we decided to focus on specific gene sets (Molecular Signatures DB-MSigDB) related to CD8+ T cell function and differentiation, such as GSEA26495 and GSEA41867. This supervised analysis showed that IL-17RA KO CD8+ T cells were significantly enriched in six gene sets in which the most enriched CD8+ T cell gene sets in the significance order (size of FWER *P* values) were PD1^hi^ vs. PD1^low^ (*p* < 0.001) and Naïve vs. PD1^low^ (*p* < 0.001) (Figure 3B). These results denoted that CD8+ T cells derived from IL-17RA KO mice infected with *T. cruzi* showed a phenotype with characteristics of dysfunctional and, surprisingly, naïve cells.

We then analyzed the expression patterns of genes within the categories relevant for CD8+ T cell biology and IL-17RA signaling. Direct comparison of the gene expression profiles of CD8+ T cells from infected WT and IL-17RA KO mice after normalization of the date to gene expression in cell from non-infected mice revealed that absence of IL-17 expression resulted in important differencesin the transcriptional profile of CD8+ T cells (Figure 3C). Indeed, genes encoding activation and effector cell markers, such as the KLRG family molecules CD11c and CD69, showed a completely opposite pattern of expression in CD8+ T cells from infected IL-17RA KO vs. WT mice. Furthermore, CD8+ T cells from infected IL-17RA KO mice showed clearly increased expression of genes encoding inhibitory and death receptors, including *Ctla4, Havcr2* (Tim3)*, Pdcd1* (PD1), *Tigit*, and *Fas*. We also analyzed how the absence of IL-17RA signaling affected the expression of genes encoding transcription factors (TFs) that regulate CD8+ T cell fate. Of note, there was a group of TF-encoding genes whose expression was significantly higher in CD8+ T cells from infected IL-17RA KO mice than in those of their WT counterparts. This group included genes such as *Foxo1, Irf4*, and *Foxo3* that are critical for the generation of effector CD8+ T cells (30, 31). Also, we determined that CD8+ T cells from infected WT and IL-17RA mice showed differences in the expression of many genes encoding effector molecules, cytokine receptors, homing and migration molecules, and apoptosis mediators, thus further supporting the fact that absence of IL-17RA influences CD8+ T cell fate. Finally, analysis of the gene expression of molecules associated to IL-17RA signaling (6) evidenced significant differences between CD8+ T cells from infected WT and IL-17RA KO mice. Thus, CD8+ T cells from WT mice showed increased amounts of transcripts encoding MMP-13, CCL-20, and IL-6 among others while CD8+ T cells from IL-17RA KO mice showed higher amounts of transcripts encoding TNF, IKBKb, CXCL-2, S100a8, MMP-8, and others (Figure 3D).

To broadly analyze the significance of the transcriptional differences between CD8+ T cells from infected IL-17RA KO mice and their WT counterparts, we performed an Ingenuity Pathway Analysis (IPA) on genes marked in red in Figure 3A. The genes were up-regulated in infected CD8+ T cells from both groups of mice, but were expressed at significantly higher levels in CD8+ T cells from IL-17RA KO mice. The top 20 “Diseases and Bio-functions,” “Canonical pathways,” and “Upstream regulators” that were most significantly altered in CD8+ T cells from IL-17RA KO mice infected with *T. cruzi* are shown in Figures 3E–G. Interestingly, “Cell death,” “Apoptosis of Lymphocytes” bio-functions (Figure 3E) and the “p53 Signaling” (Figure 3F) canonical pathway emerged as significantly up-regulated. These results support the notion that the lack of IL-17RA signaling during *T. cruzi* infection significantly affected the survival and senescence status of CD8+ T cells, as well as various biological features. Finally, to elucidate which molecule/s were involved in the observed differences in gene expression, we performed an “upstream regulator analysis” (Figure 3G). Many modulators of the inflammatory responses, including the cytokines IL-2 and IL-6 and the NF-κB regulators CHUK and IKBKG, were significantly up-regulated in CD8+ T cells from infected IL-17RA KO mice. Conversely, the genes for MKL-2 and MKL-1, which interact with transcriptional regulator serum response factor, were down-regulated. Notably, IRF4, and to a lesser extent other TFs such as BATF, STAT1, and AHR, emerged as potential upstream regulators of the transcriptional program induced by *T. cruzi* infection in CD8+ T cells from IL-17RA KO mice.

### Absence of IL-17RA signaling during *T. cruzi* infection results in CD8+ T cell exhaustion

**Figure 4:**
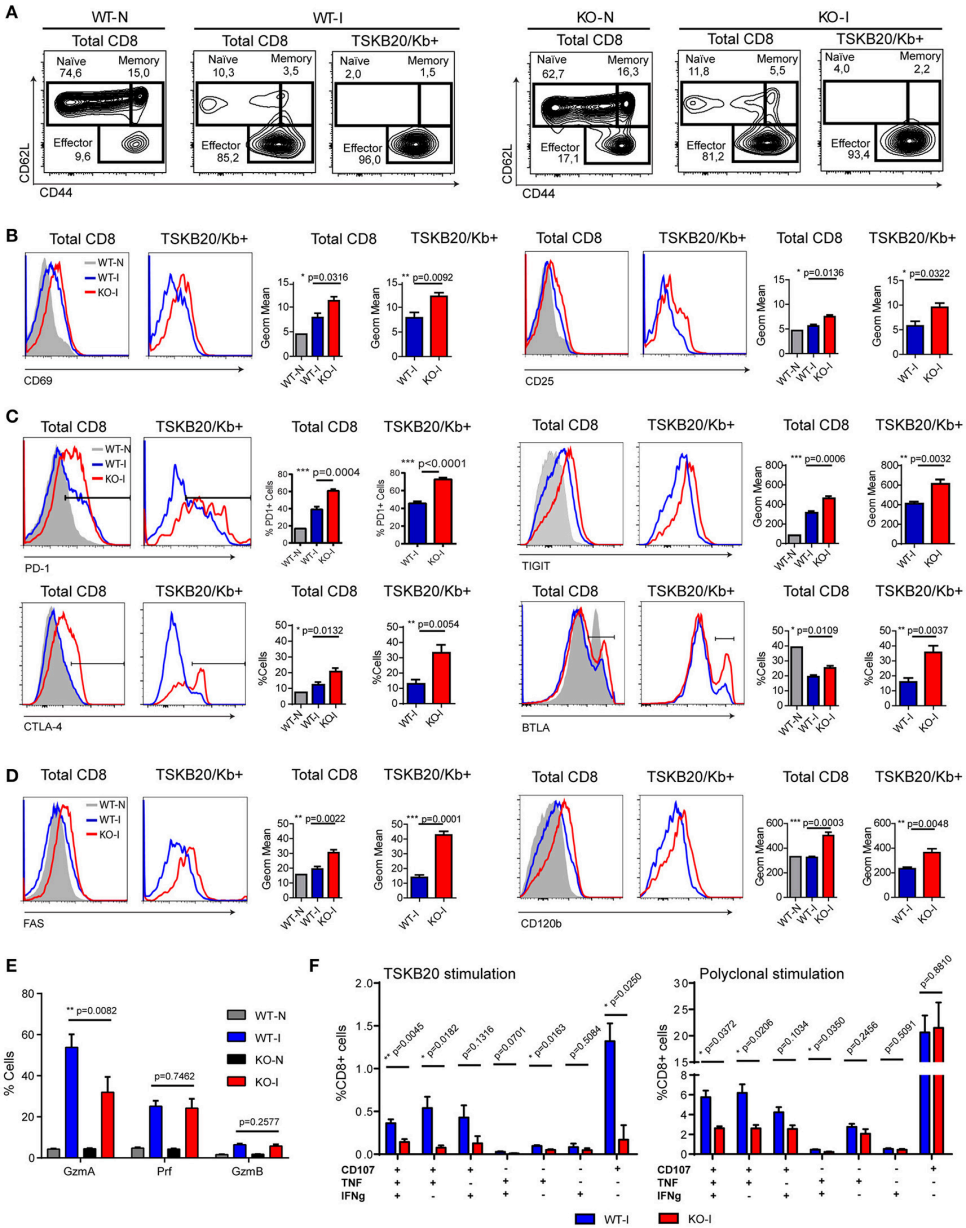
CD8+ T cells from *T. cruzi* infected IL-17RA KO mice show phenotypic and functional features compatible with exhausted cells. Representative flow cytometry data that show the phenotype of total and TSKB20/Kb+ CD8+ T cells from the spleen of infected WT (WT-I) and IL-17RA (KO-I) mice (22 dpi). Stainings of non-infected WT (WT-N) and KO (KO-N) mice are showed in plots or gray tinted histograms for comparison. **(A)** Plots of CD62L vs. CD44 expression. Gates indicate the frequency of naïve, memory and effector subsets. **(B–D)** Representative histograms and statistical analysis of the geometric mean of expression or percentage of cells that express activation markers **(B)**, inhibitory receptors **(C)**, and death receptors **(D)** in total and TSKB20/Kb+ CD8+ T cells from the spleen of infected WT (WT-I) and IL-17RA (KO-I) mice (22 dpi). Data in statistical analysis **(B–D)** are presented as mean ± *SD, N* = 4−6 mice. **(E)** Percentage of CD8+ T cells from the spleen of non-infected (N) and 22-day infected (I) WT and KO mice expressing Granzyme A (GzmA), Perforin (Prf) or Granzime B (GzmB) after 5 h of PMA/Ionomycin stimulation. Data shown as mean ± *SD, N* = 2−5 mice. **(F)** Percentage of CD8+ T cells from the spleen of 22-day infected WT and KO mice that exhibit different combinations of effector functions including CD107a mobilization and/or IFNγ and/or TNF production upon 5 h of the indicated stimulation. Data shown as mean ± *SD, N* = 5 mice. Data were background subtracted. All *P* values were calculated using two-tailed *T* test. Data are representative of two-three independent experiments.

Given the results obtained from the microarray analysis, we performed a phenotypic characterization of the CD8+ T cells from the different experimental mouse groups. Analysis of CD62L vs. CD44 expression showed that upon *T. cruzi* infection, more than 80% of the total CD8+ T cells from the spleen of infected WT and IL-17RA KO mice acquired an activated/effector phenotype characterized by CD62L^low^CD44^hi^ expression (Figure 4A). Within the TSKB20-specific population, the effector cells constituted more than 95% of the total number of cells. These results highlighted that CD8+ T cell activation triggered by *T. cruzi* infection is not compromised in the absence of IL-17RA. Indeed, total and parasite-specific CD8+ T cells from the infected IL-17RA KO mice had significantly elevated expression of prototypical activation markers such as CD69 and CD25 in comparison to that of CD8+ T cells from WT mice (Figure 4B), which corroborated the results from our microarray analysis.

We next determined the expression of inhibitory and death receptors in CD8+ T cells from WT and IL-17RA KO mice infected with *T. cruzi*. The total and TSKB20-specific CD8+ T cells from infected WT mice had an increased frequency of PD-1+ and CTLA-4+ cells, slightly higher TIGIT expression, and reduced frequency of BTLA+ cells compared to that of non-infected mice (Figure 4C). Notably, total and TSKB20-specific CD8+ T cells from infected IL-17RA KO mice showed significantly increased frequency of PD-1+, CTLA-4+ and BTLA+ cells as well as higher expression of TIGIT. Furthermore, these cells presented an apoptotic phenotype characterized by the expression of high levels of CD95/Fas and CD120b/TNF-R2 (Figure 4D). Total and TSKB20-specific CD8+ T cells from infected IL-17A/IL-17F DKO mice showed a profile of expression of inhibitory (PD-1) and death receptors (Fas/CD95) that was comparable to that of total and TSKB20-specific CD8+ T cells from infected IL-17RA KO mice (Supplementary Figures 1C,D).

Further phenotypic characterization included effector molecules involved in cytotoxicity such as Granzyme A (GzmA), Granzyme B (GzmB), and Perforin-1 (Prf). As GzmB and Prf were barely detectable *ex vivo* (data not shown), the expression of the three cytotoxic molecules was determined in splenocytes from non-infected and infected WT and IL-17RA KO mice after 5 h of stimulation with PMA/ionomycin (Supplementary Figure 4A). In agreement with the microarray data, we determined that CD8+ T cells from infected IL-17RA KO mice had significantly reduced expression of GzmA in comparison to their counterparts from infected WT mice, whereas the expression levels of GzmB and Prf were similar between both groups (Figure 4E).

To address whether the phenotypic features of CD8+ T cells elicited in absence of IL-17RA correlated with altered functionality, we compared the effector function of CD8+ T cells from infected WT and IL-17RA KO mice. To this end, we analyzed the mobilization of CD107a and secretion of effector cytokines upon specific and polyclonal activation *in vitro*. Stimulation of splenocytes from infected IL-17RA KO mice with the TSKB20 peptide resulted in a significant reduction of the frequency of CD8+ T cells with polyfunctional characteristics (i.e., surface CD107a expression and IFNγ and/or TNF production) in comparison to WT control mice (Figure 4F, left panel and Supplementary Figure 3). Although a poor response was anticipated due to the low frequency of TSKB20-specific CD8+ T cells in infected IL-17RA KO mice, the antigen-specific effector response of CD8+ T cells was not significantly different from background, suggesting the presence of both quantitative and qualitative defects. Polyclonal stimulation with PMA/ionomycin increased the percentage of CD8+ T cells from infected IL-17RA KO mice that had a polyfunctional response, although the magnitude of this effector response was significantly lower than that of WT mice (Figure 4F, right panel and Supplementary Figure 3).

To determine if the higher parasite burden in infected IL-17RA KO mice may underlie the upregulation of inhibitory receptors in CD8+ T cells from these mice, we evaluated the phenotype of CD8+ T cells from WT mice infected with increasing parasite doses. Interestingly, increasing the load of *T. cruzi* did not diminish the frequency of parasite-specific CD8+ T cells, nor did it promote the upregulation of the inhibitory and death receptors evaluated (Supplementary Figures 4B–D).

### Checkpoint blockade partially restores parasite-specific CD8+ T cell immunity and enhances parasite control in infected IL-17RA KO mice

**Figure 5:**
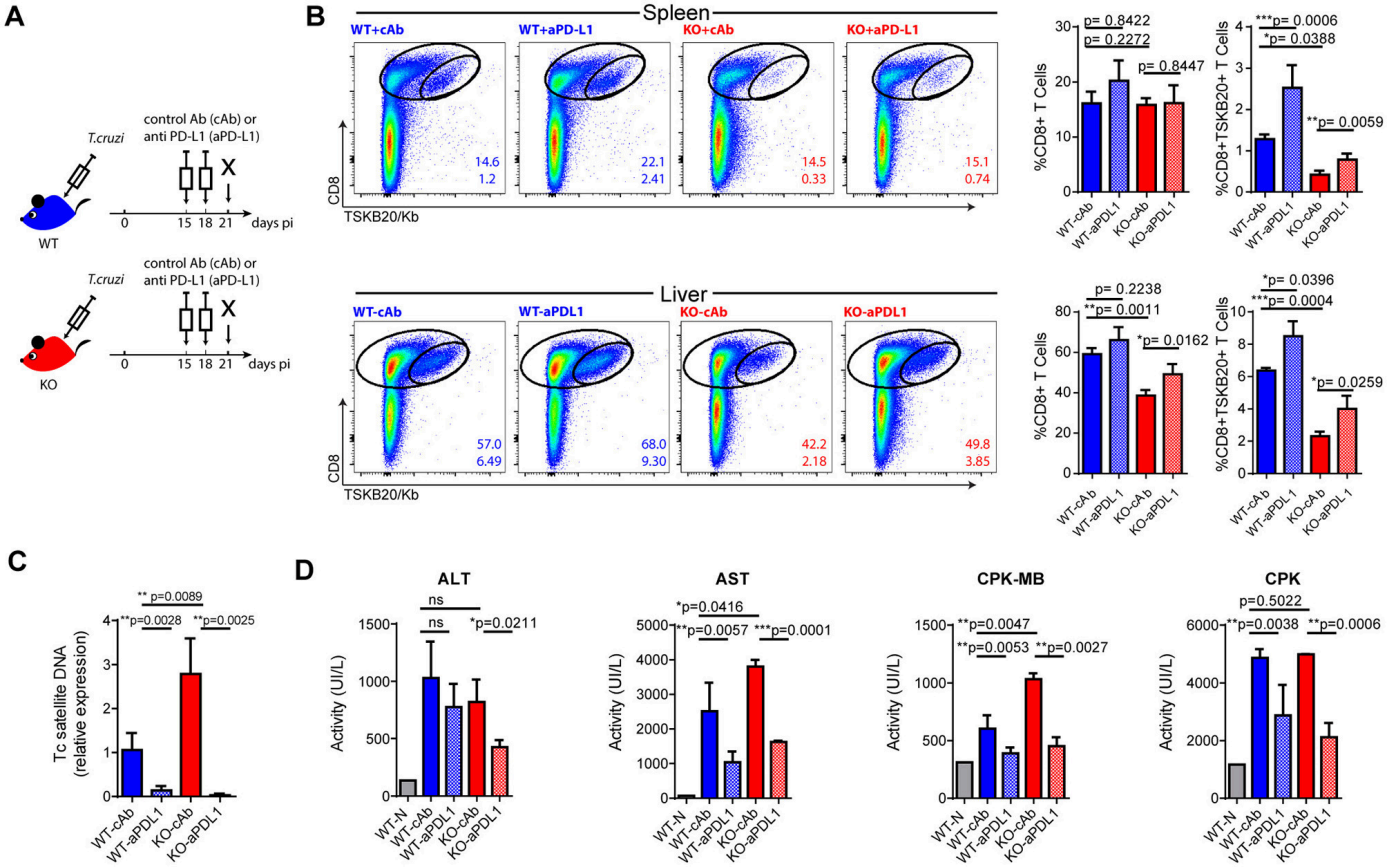
Checkpoint blockade reinvigorates parasite-specific CD8+ T cell immunity and enhances parasite control in infected IL-17RA KO mice. **(A)** Layout of the checkpoint blockade experiment. **(B)** Representative plots and statistical analysis of CD8 and TSKB20/Kb staining in spleen and liver of the indicated experimental groups. Numbers within plots indicate the frequency of total CD8+ cells (up) and TSKB20-specific CD8+ T cells (down) at 21 dpi. Bar graphs represent data as mean ± *SD, N* = 7 mice. **(C)** Relative amount of *T. cruzi* satellite DNA in the spleens of the indicated experimental groups at 21 dpi. Murine GAPDH was used for normalization. Data are presented as mean ± *SD* relative to WT-cAb group, *N* = 7 mice. **(D)** Enzymatic activity (UI/L) of alanine transaminase (ALT), aspartate transaminase (AST), creatinine kinase-MB and creatinine phosphokinase (CPK) on plasma of the indicated experimental groups at 21 dpi. *N* = 7 mice. All *P* values were calculated with two-tailed *T* test. Data are representative of two independent experiments.

CD8+ T cell exhaustion has been associated with poor microbial control during infection with various pathogens, as well as with tumor progression in cancer. In these settings, a checkpoint blockade has been shown to efficiently restore the magnitude and functionality of effector T cells and minimize damage from the insult (32). Furthermore, a PD-L1 blockade has been effective in preventing T cell apoptosis in murine and human sepsis models (33, 34). Our results highlight that the absence of IL-17RA signaling during *T. cruzi* infection leads to poor CD8+ T cell effector function and parasite persistence. This prompted us to evaluate whether a checkpoint blockade could restore a resistance phenotype to infected IL-17RA KO mice. As PD-1 is highly expressed on parasite-specific CD8+ T cells from infected IL-17RA KO mice, we targeted this checkpoint receptor by injecting blocking anti-PD-L1 Abs at 15 and 18 dpi, before the contraction of a parasite-specific CD8+ T cell response (Figure 5A). We determined that PD-L1 blockade in infected IL-17RA KO significantly augmented the magnitude of the TSKB20-specific CD8+ T cell response by 21 dpi, particularly in the spleen and liver (Figure 5B). A similar response to treatment was observed in infected WT mice. Notably, the enhanced *T. cruzi*-specific CD8+ T cell immunity was correlated with a marked reduction in the levels of tissue parasitism (Figure 5C). In addition, inhibition of PD-L1 also lead to a milder infection, as highlighted by the significantly reduced levels of several damage biomarkers, such as the transaminase aspartate aminotransferase (AST), creatine kinase total (CK), and myocardial band (CK-MB) (Figure 5D). The rescue effects of the PD-L1 checkpoint blockade during *T. cruzi* infection, namely parasite persistence and tissue damage, were more impressive in IL-17RA KO mice than in WT mice, as they showed extremely high susceptibility to *T. cruzi* infection in the absence of treatment.

### Intrinsic IL-17RA signaling modulates the maintenance and phenotype of CD8+ T cells activated during *T. cruzi* infection

**Figure 6:**
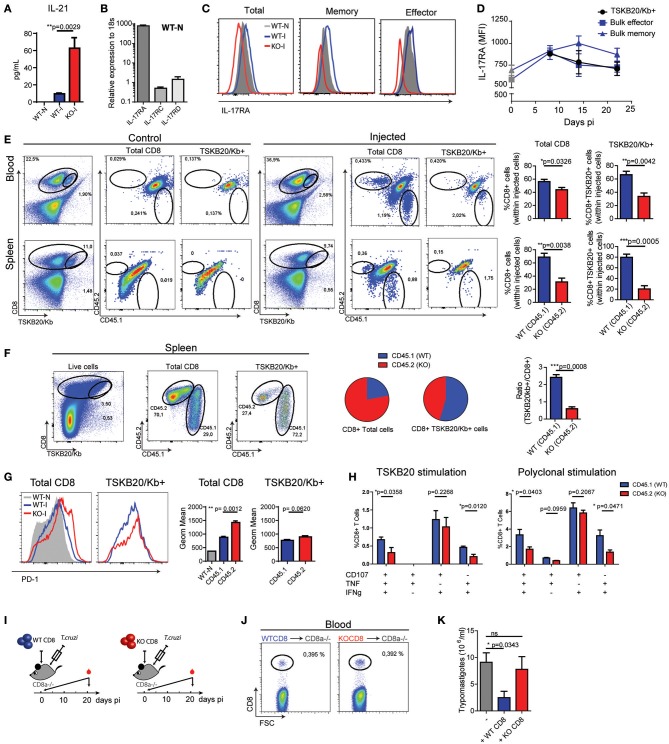
Intrinsic IL-17RA-signaling modulates the maintenance, phenotype and function of CD8+ T cells during *T. cruzi* infection. **(A)** Concentration of IL-21 in plasma of infected (20 dpi) WT and IL-17RA KO mice. Data are presented as mean ± *SD, N* = 4 mice. **(B)** Amounts of *Il17ra, Il17rc*, and *Il17rd* transcript determined in CD8+ T cells purified from the spleen of non-infected WT mice, normalized to 18S RNA. Data are presented as mean ± *SD, N* = 4 mice **(C)** Representative histograms of the expression of IL-17RA (protein) on total, memory and effector CD8+ T cell subsets defined according to CD44 and CD62L staining in spleen cell suspensions from non-infected (N) and infected (I) WT mice (14 dpi). Staining of IL-17RA on spleen CD8+ T cells from IL-17RA KO mice is showed as negative control. **(D)** Kinetic evaluation of the upregulation of IL-17RA (MFI: mean of fluorescence intensity) induced by *T. cruzi* expression on bulk effector and memory CD8+ T cells and TSKB20-specific CD8+ T cells. Data in statistical analysis are presented as mean ± *SD, N* = 4. **(E)** Representative plots and statistical analysis of CD8 and TSKB20/Kb staining and of CD45.1 and CD45.2 staining within Total and TSKB20/Kb+ CD8+ T cells in the blood and spleen of infected CD45.1/CD45.2 WT recipient mice (20 dpi) non-injected (control) or injected with equal numbers of CD45.1+ WT and CD45.2+ KO CD8+ T cells. Numbers in the plots indicate the frequency of the correspondent cell subset. Bar graphs display the frequency of CD45.1+ WT cells and CD45.2+ KO cells within the indicated populations upon gating only in the injected cells. **(F)** Representative plots and statistical analysis of CD8 and TSKB20/Kb staining and of CD45.1 and CD45.2 staining within Total and TSKB20/Kb+ CD8+ T cells in the spleen of infected CD8α-/- recipients (17 dpi) injected with equal numbers of CD45.1+ WT and CD45.2+ KO CD8+ T cells. Pie charts display the frequency of CD45.1+ WT and CD45.2+ KO cells within the indicated gates. Bar graph shows the ratio between the frequencies of TSKB20/Kb+ CD8+ T cells and the total CD8+ T cells within CD45.1 WT and CD45.2 IL-17RA KO populations. **(G)** Representative histograms and statistical analysis of the geometric mean of PD-1 expression in total and TSKB20/Kb+ CD8+ T cells within CD45.1+ WT and CD45.2+ IL-17RA KO CD8+ T cells from the spleen of CD8α-/- recipient mice. Data in statistical analysis are presented as mean ± *SD, N* = 4−6 mice. **(H)** Percentage of polyfunctional effector cells denoted by expression CD107a, IFNγ and/or TNF upon 5 h of the indicated stimulation on CD45.1+ WT and CD45.2+ IL-17RA KO CD8+ T cells from the spleen of CD8α-/- recipient mice. Data shown as mean ± *SD, N* = 4 mice. Data were background subtracted. All *P* values in **(E–G)** were calculated using two-tailed *T* test. Data are representative of two independent experiments. **(I)** Experimental layout of the adoptive transfer of equal number of CD8+ T cells purified from WT mice or IL-17RA KO mice into CD8α knockout mice immediately followed by *T. cruzi* infection. *N* = 3 mice/group. **(J)** Representative dot plots of the frequency of CD8+ T cells determined 1 day after injection in blood of the different experimental groups. **(K)** Parasitemia (15 dpi) in the different experimental groups. Infected non-transferred CD8α knockout mice were used as control. *P* value calculated using Gehan-Breslow-Wilcoxon Test. Data in **(A–J)** are representative of two independent experiments.

To understand if IL-17RA plays a direct role in the modulation of CD8+ T cell responses to *T. cruzi*, we first quantified the serum concentration of IL-21, a cytokine known to modulate CD8+ T cell immunity (35). Of note, we determined that in comparison to infected WT mice, infected IL-17RA KO mice displayed higher expression of IL-21 (Figure 6A). This result highlights that increased expression of IL-21 is not sufficient to compensate for the lack of IL-17RA signaling via restoring CD8+ T cell responses during *T. cruzi* infection.

We next evaluated the possibility of a direct effect of IL-17RA signaling in the modulation of CD8+ T cell responses, given that IL-17RA is upregulated in response to cytokines that boost CD8+ T cell responses such as IL-21 (26, 36). The IL-17RA subunit interacts with IL-17RC to form the functional receptor for both IL-17A and IL-17F [reviewed in Gaffen 6). Recently, also IL-17RD was shown to bind to IL-17RA to differentially regulate IL-17A-inducing signaling pathways (37, 38). Therefore, we evaluated the mRNA levels of IL-17RA, IL-17RC, and IL-17RD by quantitative PCR. In agreement with published data, mRNA encoding IL-17RA were detected in splenic CD8+ T cells (Figure 6B). Transcripts encoding *Il17rc* and *Il17rd* were also detected in these cells, but at remarkably lower levels in comparison to *Il17ra*. Studies by flow cytometry confirmed the expression of IL-17RA on CD8+ T cells. Notably, IL-17RA expression varied depending on the CD8+ T cell subset, and was higher in memory cells defined as CD44^hi^CD62L^hi^ than in the effector (CD44^hi^CD62L^lo^) and naïve (CD44^lo^CD62L^hi^) subsets (Figure 6C). *T. cruzi* infection upregulated IL-17RA expression in CD8+ T cells, to levels and kinetics specific for each subset (Figures 6C,D). We observed that IL-17RA expression is substantially upregulated in bulk memory CD8+ T cells during *T. cruzi* infection, but is only transiently increased in bulk effector CD8+ T cells at early time points (Figure 6D). As expected given their effector nature, TSKB20-specific CD8+ T cells expressed IL-17RA at a level that was comparable to bulk effector cells.

To directly address the impact of intrinsic IL-17RA signaling in the development of CD8+ T cell responses during *T. cruzi* infection, we performed a series of adoptive transfer experiments. First, we transferred an equal number of congenically marked IL-17RA-deficient (CD45.2+) and WT (CD45.1+) CD8+ T cells into WT (CD45.1+/CD45.2+) recipient mice. Recipient mice were infected, and the presence of injected cells in the total and TSKB20-specific CD8+ T cell pools was examined at 20 dpi. As expected, no CD45.2+ KO or CD45.1+ WT CD8+ T cells were detected in infected F1 (CD45.1+/CD45.2+) WT hosts that were not injected (control mice). When analyzing the competitive adoptive transfer experiment, we observed that within the population of injected cells, IL-17RA KO CD8+ T cells were outcompeted by their WT counterparts in both total and parasite-specific CD8+ T cell subsets in all the organs examined, including blood and spleen (Figure 6E).

To overcome the possible limitations derived from the presence of endogenous WT CD8+ T cells in lymphoreplete hosts, we repeated the competitive adoptive transfer experiment in CD8α-/- mice. At 17 dpi, the frequency of IL-17RA KO cells was double that of WT cells within the total population of CD8+ T cells (Figure 6F), likely as result of increased homeostatic proliferation of polyclonal IL-17RA KO CD8+ T cells (Figure 2A). In contrast, WT cells significantly outnumbered IL-17RA KO cells within the TSKB20-specific CD8+ T cell pool, indicating that intrinsic expression of IL-17RA provides a maintenance advantage for parasite-specific CD8+ T cells. Indeed, the ratio between the percentage of WT CD8+ T cells within TSKB20-specific gate and the polyclonal total CD8+ T cells was significantly higher than that of IL-17RA KO CD8+ T cells (Figure 6F). Phenotypic evaluation revealed that IL-17RA KO CD8+ T cells exhibited higher expression of the inhibitory receptor PD-1 (Figure 6G). Upon antigen specific stimulation with TSKB20, WT CD8+ T cells exhibited a significantly higher polyfunctional effector response in comparison to IL-17RA KO CD8+ T cell from the same host (Figure 6H, left panel and Supplementary Figure 5). An identical result was observed after polyclonal stimulation (Figure 6H, right panel and Supplementary Figure 5).

Finally, we aimed to address the relevance of CD8+ T cell-intrinsic IL-17RA signaling for host protection during *T. cruzi* infection. To this end, we evaluated the ability to control parasite replication in CD8α-/- mice transferred with IL-17RA KO CD8+ T cells in comparison to CD8α-/- mice transferred with WT CD8+ T cells (Figure 6I). As a control, we determined that the frequency of circulating CD8+ T cells at day one post-injection was the same between both groups of mice (Figure 6J). CD8α-/- mice were extremely susceptible to *T. cruzi* infection and displayed uncontrolled parasitemia at day 21, even with an infection dose of 100 trypomastigotes, which was 50 times less than the dose used in previous experiments. Furthermore, all these mice succumbed by day 25 post-infection (data not shown). Remarkably, the transfer of IL-17RA-sufficient WT CD8+ T cells, but not of IL-17RA-deficient KO CD8+ T cells, was able to enhance parasite control and significantly reduce parasitemia (Figure 6K).

## Discussion

During the last few decades, many reports have delineated the immunological mechanisms underlying IL-17-mediated roles in host protection against infection as well as in inflammatory diseases (39, 40). In this study, we uncover a novel mechanism by which IL-17RA signaling regulates CD8+ T cell responses and promotes protection against a protozoan infection. Our findings provide new elements to elucidate the cytokines and pathways leading to robust CD8+ T cell responses against infections with intracellular microbes and consequently, may have important implications for vaccine and therapy design.

The integral role of the IL-17 family in host defense against extracellular bacterial and fungal pathogens has been well established, and recent studies have implicated the family of genes in potentiating immunity against intracellular bacteria, viruses, and parasites (3). So far, the IL-17-dependent protective mechanisms were believed to be dependent on the activation of both non-immune and innate immune cells to sustain inflammation (8). Notably, the IL-17 family of proteins only indirectly promotes adaptive cellular immune responses (41, 42). In this regard, there is evidence to suggest the role of IL-17 cytokines in sustaining specific CD8+ T cell responses that promote host resistance to *Listeria* infection and melanoma (43, 44). In these settings, the IL-17-mediated induction of protective CD8+ T cells depended on enhanced recruitment of cross-presenting dendritic cells, although direct effects on CD8+ T cells were not ruled out. Very recently, IL-17 was shown to directly potentiate CD8+ T cell cytotoxicity against West Nile Virus infection (9). In line with this, we demonstrate that mice deficient in IL-17RA showed an abortive CD8+ T cell response during *T. cruzi* infection characterized by an early reduction in the number of parasite-specific CD8+ T cells. Concomitantly, infected IL-17RA KO mice showed poor control of the parasite in target tissues such as spleen, liver, and heart. By using different experimental approaches, including detailed phenotypic, functional and transcriptional profiling of CD8+ T cells from IL-17RA KO mice infected with *T. cruzi*, we demonstrated that CD8+ T cell-intrinsic IL-17RA signaling is required to sustain CD8+ T cell maintenance and pathogen-specific CD8+ T cell immunity, particularly during the expansion phase.

Whether one or more of the four cytokines that signal through IL-17RA is involved in these events is currently unknown. However, our results led us to postulate that IL-17A is the main cytokine involved in the IL-17RA-mediated effect of CD8+ T cells. This postulate is based on the marked similarities in the phenotype of the infected IL-17A/F DKO and IL-17RA KO mice and the significant consequences of the IL-17A neutralization in mice and IL-17A supplementation in CD8+ T cell cultures. Still, a possible role of other members of this family are not ruled out given that at least two other the IL-17RA signaling cytokines besides IL-17A (i.e., IL-17F and IL-17E/IL-25) are increased during *T. cruzi* infection (4). Regarding receptor expression, we determined that CD8+ T cells express the transcripts encoding not only IL-17RA but also IL-17RC and IL-17RD. Of note, we determined that *Il17ra* transcripts are present in significantly higher amounts than *Il17rc* and *Il17rd*. These findings are noteworthy as high levels of IL-17RA seem to be required for effective responses to IL-17 (45), and also because they also pose the question of whether IL-17RC or IL-17RD are effectively expressed on CD8+ T cells and required for IL-17 signal transduction. Although, the receptor complex formed by IL-17RA and IL-17RC is considered essential for IL-17 signal transduction (46), some studies reported that hematopoietic cell types (including T cells) show IL-17-dependent responses even in the absence of IL-17RC (47–49). It should be further established whether IL-17RD may interact with IL-17RA and replace IL-17RC to mediate the IL-17 signaling in these populations as previously reported (37). In the same direction, to dissect the precise signaling requirement for IL-17 signal transduction in CD8+ T cells may provide important information in terms of the IL-17 biology.

Besides a direct role of IL-17 cytokines, it is possible that a crosstalk of IL-17 with other cytokines that regulate CD8+ T cell fate such as IL-21 might be indirectly involved in the observed qualitative defects. In this regard, Hoft and colleagues described that TCR-transgenic CD4+ T cells specific for an immunodominant peptide of *T. cruzi* that were polarized *in vitro* into Th17 cells could potentiate CD8+ T cell immunity through a mechanism that is IL-21-dependent, but IL-17-independent (50). It remains to be established whether the *in vitro* generated Th17 cells are equivalent to those differentiated *in vivo* within the host during *T. cruzi* infection. Indeed, we determined that infected IL-17RA KO mice displayed higher concentrations of serum IL-21 than infected WT control mice, suggesting that the altered CD8+ T cell response observed in absence of IL-17RA signaling is unlikely to be a result of deficient IL-21 production.

CD8+ T cells from infected IL-17RA KO mice showed increased apoptosis and a transcriptional landscape associated with canonical cell death and senescence pathways. In addition, these cells exhibited high expression of multiple inhibitory and death receptors that correlated with reduced *ex vivo* effector responses and a gene signature that positively correlated with those defined for dysfunctional PD-1^high^ CD8+ T cells (51, 52). Altogether, these features partially resembled those of dysfunctional CD8+ T cells described in many chronic infections and cancer that are characterized by the progressive loss of T cell function, sustained expression of inhibitory receptors, and high susceptibility to deletion (53). Interestingly, we found that the functional CD8+ T cell response observed in *T. cruzi*-infected WT mice [this manuscript and (54)] was significantly impaired in the absence of IL-17RA signaling. In this regard, although it is conceivable that the increased tissue parasitism in IL-17RA KO mice favored CD8+ T cell exhaustion, infection of WT mice with increasing parasite loads does not induce expression of inhibitory receptors or deletion of parasite-specific CD8+ T cells, nor does it result in impaired CD8+ T cell cytotoxic effector function (55). As cytokines are essential for the regulation of exhaustion (20), we performed adoptive transfer experiments that supported the notion that IL-17 signaling influenced CD8+ T cell survival and function partially through cell intrinsic mechanisms. Interestingly, we determined that IL-17RA is expressed at different levels according to each particular CD8+ T cell subset. Specifically, IL-17RA expression was increased and sustained in memory cells, but was transiently up-regulated in activated effector cells at early stages of infection. These results, together with those showing that IL17RA downregulation is included in the gene signature of exhausted T cells (56, 57), suggest that the IL-17/IL-17RA pathway modulates CD8+ T cell fate, thus preventing cell exhaustion in activated cells and sustaining long-lasting cytotoxic responses. In this regard, adoptive transfer of WT, but not of IL-17RA KO CD8+ T cells, promoted parasite control in immuno-deficient CD8α-/- mice. In line with this, we demonstrated that anti-PD-L1 Abs during the CD8+ T cell expansion phase reinvigorated the parasite-specific CD8+ T cell responses in infected IL-17RA KO mice, thus restoring robust parasite control and reducing pathology to levels comparable to infected WT controls. Remarkably, we observed that a short regimen of PD-L1 neutralization increased the frequency of parasite-specific CD8+ T cells by two-fold in both treated groups, although the magnitude of the response in infected IL-17RA KO mice remained lower than in WT control mice. Given the high levels of inhibitory receptors in CD8+ T cells elicited by *T. cruzi* infection in absence of IL-17RA, it is likely that the combination of different checkpoint Abs may have a stronger effect in these settings, as reported in cancer models (58).

The role of checkpoint inhibitors during *T. cruzi* infection, particularly the PD-1/PD-L1 axis, has been understudied thus far. One report demonstrated that genetic disruption of this inhibitory pathway increased the effector immune response to favor parasite control, but also promoted cardiac pathology and compromised host resistance to the infection (59). In this context, our data provide evidence that a PD-L1 checkpoint blockade at the CD8+ T cell expansion phase not only restored resistance to *T. cruzi* absence of IL-17RA signaling, but also boosted protective CD8+ T cell immunity during the natural infection. These findings are relevant to a growing area of research aimed at investigating the therapeutic potential of immune checkpoint pathways during chronic infections (60). In recent years, several reports have shown that immune cell exhaustion or dysfunction is a common finding in human Chagas disease, particularly in patients with severe symptoms (61–64). There is certain consensus that even very low parasite loads in tissues may drive, in the long term of the human infection (two decades), the expression of inhibitory receptors and cell dysfunction in T cells. Consequently, this likely drives an immune modulatory mechanism and reduced tissue damage in the later stages of infection. Interestingly, many reports have described that patients with moderate to severe chronic chagasic cardiomyopathy have reduced production of IL-17, supporting a protective role of the cytokine against cardiac damage (65, 66). Altogether, previous findings and those reported here support the hypothesis that elevated IL-17 production in the context of persistent parasite levels may prevent T cell dysfunction and the associated immune imbalance that cause chronic myocarditis. Given the relevance for the understanding of the immunopathology during human Chagas disease, this hypothesis merits further investigation in future studies.

The comparison of the transcriptome of CD8+ T cells from infected IL-17RA KO and WT mice supported the notion that IL-17RA signaling influences the transcriptional program of effector CD8+ T cells. These conclusions were based on the transcriptome of bulk CD8+ T cells and, consequently, may present some caveats as result of the heterogeneity of the sample and the “dilution” of the parasite-specific response. However, we determined that during *T. cruzi* infection most CD8+ T cells acquire an activated phenotype. Furthermore, total and TSKB20-specific CD8+ T cells from infected WT and IL-17RA KO mice exhibit similar expression of activation and differentiation markers and inhibitory and death receptors. Moreover, despite the fact that TSKB20-specific CD8+ T cells were the immunodominant population within effector cells, CD8+ T cells specific for other parasite peptides (i.e., TSKB18) were also detectable in infected WT and IL-17RA KO mice (data not shown). Accordingly, we consider that transcriptomic profiling of bulk CD8+ T cells activated during *T. cruzi* infection, though with some limitations, provides meaningful evidences about the role of IL-17RA signaling in dictating the transcriptional program of effector CD8+ T cells. Interestingly, the IPA analysis of the transcriptome of CD8+ T cells arising IL-17RA KO mice infected with *T. cruzi* highlighted IRF4 and, to a minor extent, BATF as potential upstream regulators of the IL-17RA phenotype. Notably, both TFs have been reported to be required for survival and differentiation of early effector CD8+ T cells (67–72). IRF4 promotes CD8+ T cell expansion and differentiation (69), but it also induces the expression of molecules associated with an exhausted status such as Blimp-1, CTLA-4, and IL-10 (73–75). Furthermore, BATF expression may be triggered by PD-1 ligation and associated with CD8+ T cell exhaustion during chronic viral infection in humans and mice (76). Very recently, IRF4 and BATF have been shown to form a TCR-responsive transcriptional circuit that establishes and sustains T cell exhaustion during chronic viral infection (77). Therefore, it is conceivable that sustained elevated levels of both TFs could result in the induction of cell death and exhaustion program observed in CD8+ T cells from infected IL-17RA KO mice. The link between IL-17RA signaling, expression of BATF and IRF-4, and CD8+ T cell exhaustion deserves further investigation using genetic dissection approaches.

In conclusion, our results provide evidence for a novel IL-17RA-mediated mechanism that potentiates immunity to intracellular pathogens such as *T. cruzi* by improving adaptive CD8+ T cell responses. These findings, taken together with the report showing that IL-17 controls functional competence of NK cells during a fungal infection (78), suggest that the IL-17 family is likely important for both the innate and adaptive arms of the cytotoxic response against pathogens. Although it remains to be determined whether this mechanism can be applied to host resistance mediated by IL-17 cytokines in the context of other infections, these findings have two fundamental repercussions in the field of IL-17-mediated immune responses. The first is that the prolonged targeting of the IL-17 pathways as part of an anti-inflammatory treatment during autoimmune diseases or cancer may potentially have harmful effects due to defective cytotoxic responses. The second is the notion that promoting the production of IL-17 cytokines during infection or vaccination may help to elicit stronger cytotoxic responses to fight against microbes, and eventually, tumors.

## Ethics statement

This study was carried out in accordance with the recommendations of Guide to the care and use of experimental animals (Canadian Council on Animal Care, 1993) and Institutional Animal Care and Use Committee Guidebook (ARENA/OLAW IACUC Guidebook, National Institutes of Health, 2002). The protocol was approved by the Institutional Animal Care and Use Committee (IACUC) Facultad de Ciencias Químicas, Universidad Nacional de Córdoba (Approval Number 981/15) (OLAW Assurance number A5802-01).

## Author contributions

JT designed and performed most of the experiments, analyzed data, and wrote, commented on the manuscript. CA, FF, and CR performed experiments, analyzed data and commented on the manuscript. MR, MA, and MG performed experiments and commented on the manuscript. NN performed microarray experiment. WR analyzed microarray data. EP participated in microarray design and analysis and provided funding. CM and AG participated in data analysis, commented on the manuscript and provided funding. EA supervised the research, designed experiments, wrote the manuscript, and provided funding.

### Conflict of interest statement

The authors declare that the research was conducted in the absence of any commercial or financial relationships that could be construed as a potential conflict of interest.

## Supplementary material

Supplementary Figure 1 Complementary evaluation of parasite-specific CD8+ T cell responses and tissue parasitism in *T. cruzi*-infected IL-17A/IL-17F DKO mice. **(A)** Relative amount of *T. cruzi* satellite DNA in spleen and liver of infected WT and IL-17A/IL-17F DKO mice determined at 22 dpi. Murine GAPDH was used for normalization. **(B)** Representative plots and statistical analysis of CD8 and TSKB20/Kb staining in spleen of WT and IL-17A/IL-17F DKO mice at 22 dpi. Numbers on plots represent the frequency of TSKB20/Kb+ CD8+ T cells. **(C–D)** Representative histograms and statistical analysis of the geometric mean expression of the inhibitory receptor PD-1 **(C)** and the death receptor CD95/Fas **(D)** in total and TSKB20/Kb+ spleen CD8+ T cells from WT and IL-17A/IL-17F DKO mice at 22 dpi. Gray tinted histogram show staining in CD8+ T cells from non-infected WT mice. Data in statistical analysis **(A–D)** are presented as mean ± *SD, N* = 4−6 mice. *P* values calculated with two-tailed *T* test. **(A–D)** Data are representative of at least three independent experiments.

Supplementary Figure 2 Complementary studies of proliferation and apoptosis in total and parasite-specific CD8+ T cells in WT and IL-17RA KO infected mice. **(A,B)** Representative histograms and statistical analysis of Ki-67 **(A)** and Annexin V **(B)** staining within the 7ADD- gate in total (left) and TSKB20/Kb+ (right) CD8+ T cells from the spleen of WT and IL17RA KO mice at 10 dpi **(B)** and/or 20 dpi **(A,B)**. Gray tinted histogram show staining in CD8+ T cells from non-infected (N) WT mice. Histograms are representative of one out of five mice. Numbers indicate the frequency of Ki-67+ **(A)** and Annexin V+ **(B)** cells from the corresponding colored group. Bar graphs represent data as mean ± *SD, N* = 4 mice. *P* values calculated with two-tailed *T* test. **(C)** Representative histograms of TMRE staining in total (left) and TSKB20/Kb+ (right) CD8+ T cells from the spleen of infected WT mice treated with isotype control or anti-IL-17 as described in Figure 1G. Gray tinted histogram show staining in CD8+ T cells from non-infected WT mice. Numbers indicate the frequency of TMRElow (apoptotic) cells from the corresponding colored group. Histograms are representative of one out of seven mice. Bar graphs in the statistical analysis represent data as mean ± *SD, N* = 7 mice. *P* values calculated with two-tailed *T* test. **(D)** Representative plots (*N* = 3) of TMRE and LIVE/DEAD fixable aqua staining showing the frequency of cells in early apoptosis (TMRE LIVE/DEAD) and in late apoptosis/necrosis (TMRE LIVE/DEAD^+^) within cell cultures of CD8+ T cells purified from non-infected WT and IL-17RA KO mice and activated during 24 h with coated anti-CD3 and anti-CD28 in the presence of the indicated combinations of medium, IL-17A (100 ng/mL) and camptothecin (CPT, 5 μM). **(E)** Representative histograms of the expression of Bcl-2, Bim and Bax in cultures of purified CD8+ T cells activated during 24 h with coated anti-CD3 and anti-CD28 in the presence of medium or IL-17A (100 ng/mL) as indicated. **(A–E)** Data are representative of two independent experiments.

Supplementary Figure 3 Representative flow cytometry data plots of the evaluation of CD8+ T cell effector function. **(A)** Representative plots of the expression of Granzyme A (GzmA), Perforin (Prf) and Granzyme B (GrmB) determined after 5 h of PMA/ionomicin stimulation in spleen cells from non-infected (gray dots) and 22-day infected (blue dots) WT mice and in non-infected (black dots) and 22-day infected (red dots) IL-17RA KO mice. Plots are gated within CD8+ T cells. Numbers indicate the frequency of cells expressing the corresponding effector molecule within the infected groups. Data representative of two independent experiments with *N* = 3/group. **(B)** Representative plots and analysis strategy of the frequency of spleen CD8+ T cells from infected WT and IL-17RA KO mice (22dpi) showing a combination of three and two effector function including expression of CD107a, IFNγ and/or TNF upon 5 h of culture with the indicated stimulation. Plots are representative of one out of five mice. Data are representative of two independent experiments.

Supplementary Figure 4 Increasing parasite doses did not diminish the frequency of parasite-specific CD8+ T cells cells or upregulated inhibitory receptors on CD8+ T cells. **(A)** Parasitemia at 22 dpi determined in the blood of WT mice infected with increasing doses of parasites (500, 5,000 and 50,000 tripomastigotes). **(B)** Representative plots and statistical analysis of CD8 and TSKB20/Kb staining in spleen of WT infected as described in **(A)**. **(C,D)** Statistical analysis of the geometric mean of expression of inhibitory **(C)** and death **(D)** receptors in total and TSKB20/Kb+ CD8+ T cells from WT mice infected as described in A. Data in statistical analysis are presented as mean ± *SD, N* = 4−6 mice. *P* values calculated with two-tailed *T* test. **(A–D)** Data are representative of at least 2 independent experiments.

Supplementary Figure 5 Representative flow cytometry data plots of the evaluation of CD8+ T cell effector function in mice adoptively transferred. Representative plots and analysis strategy of the frequency of CD45.1+ WT and CD45.2+ IL-17RA KO CD8+ T cells from the spleen of CD8α-/- mice adoptively transferred and infected as indicated for Figure 6E. The plots show a combination of three and two effector function including expression of CD107a, IFNγ, and/or TNF upon 5 h of culture with the indicated stimulation. Plots are representative of one out of four mice. Data are representative of two independent experiments.
