# Supplementary figures and images for: IL-17RA-Signaling Modulates CD8+ T Cell Survival and Exhaustion During Trypanosoma cruzi Infection
Source: Front Immunol. 2018 Oct 11;9:2347. doi: 10.3389/fimmu.2018.02347 (PMC6193063; doi:10.3389/fimmu.2018.02347)

Suppl Fig 1

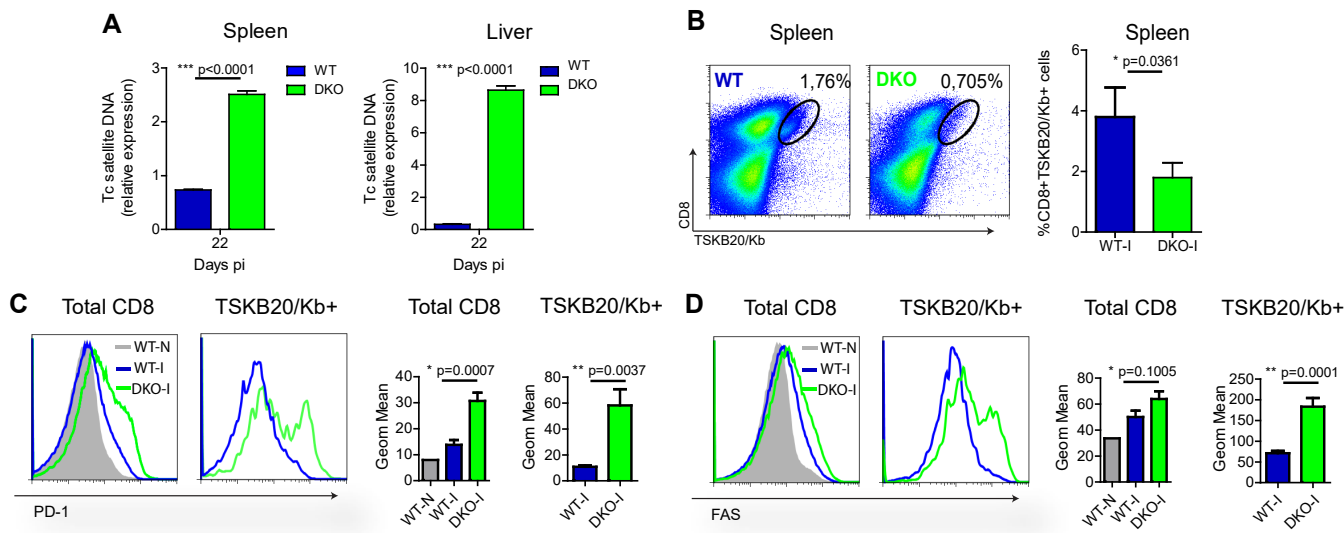

Suppl Fig 2

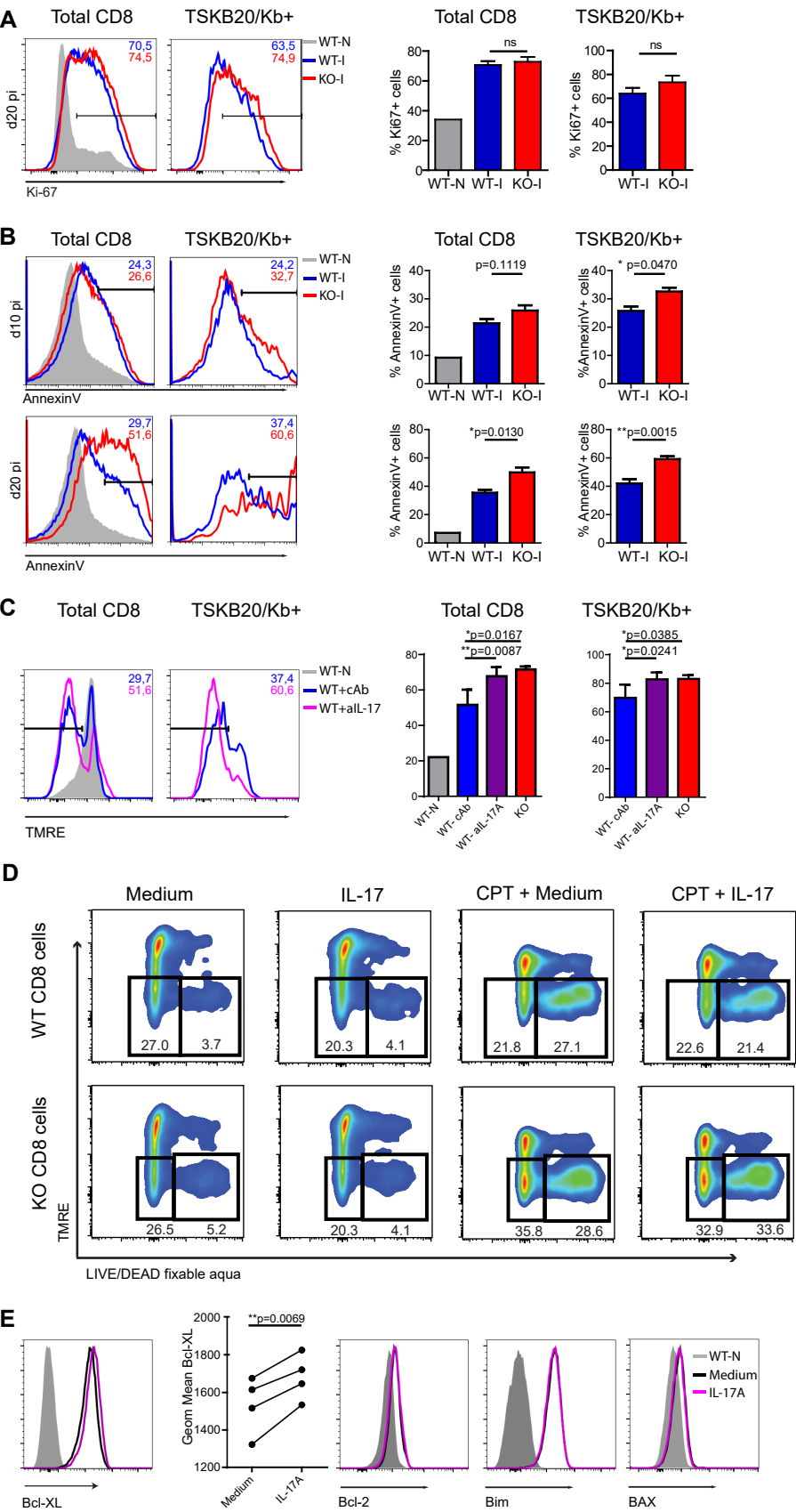

Suppl Fig 3

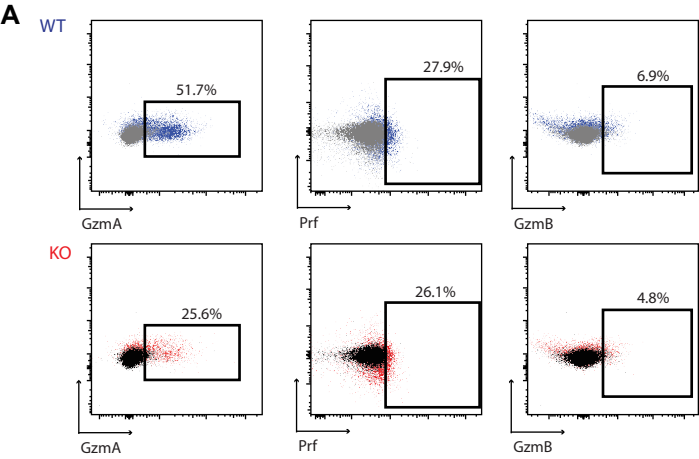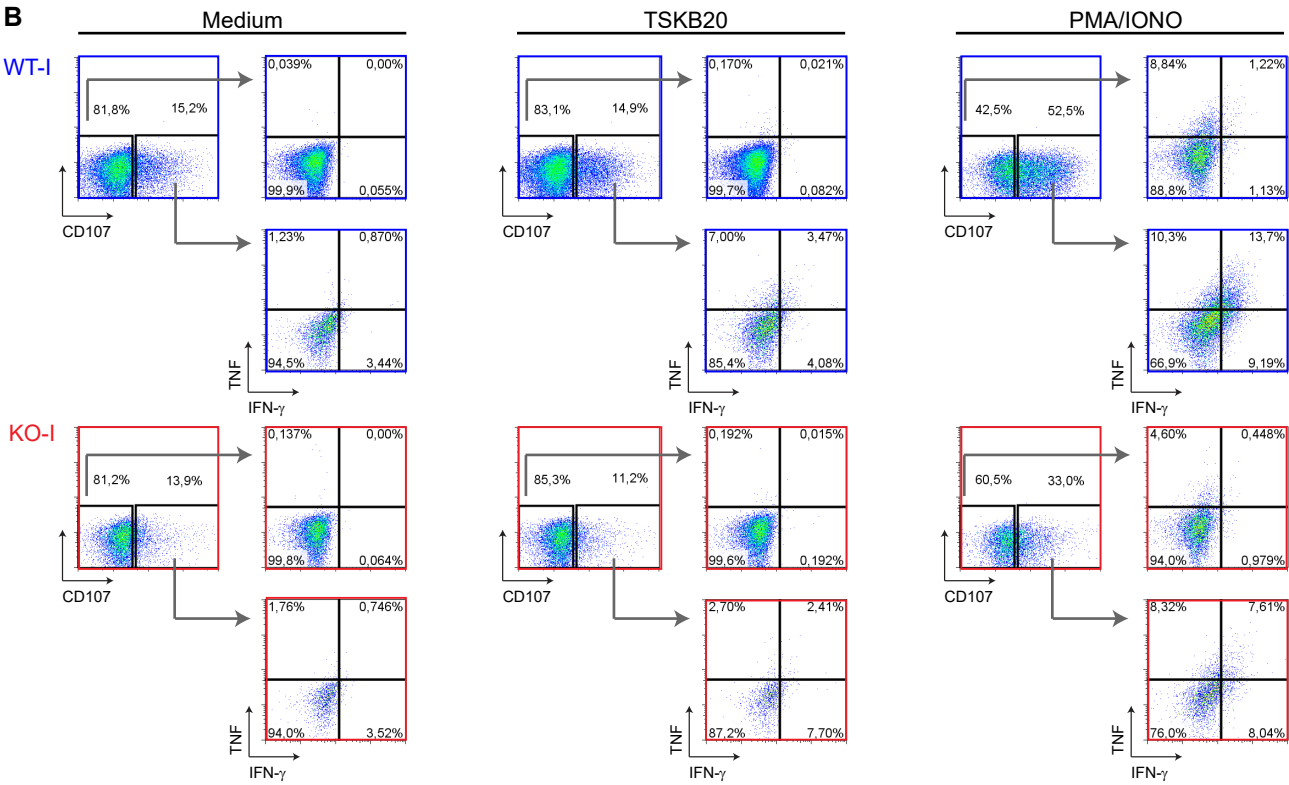

Suppl Fig 4

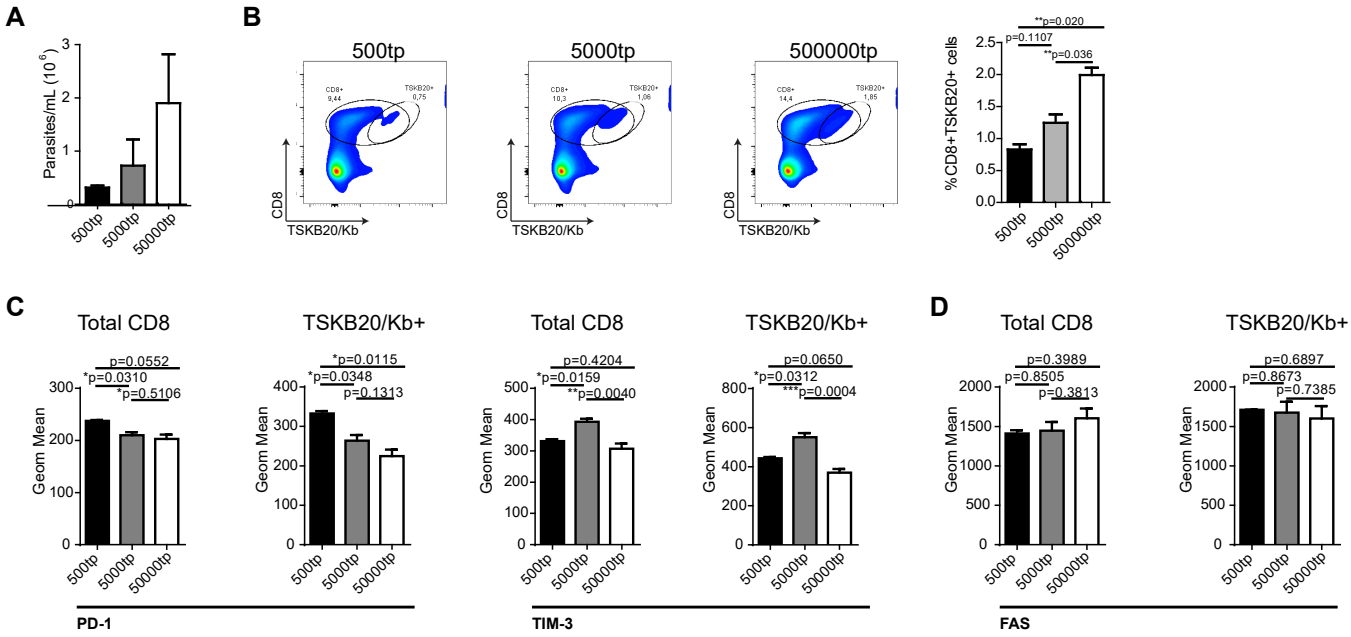

Suppl Fig 5

TSKB20

PMA/IONO

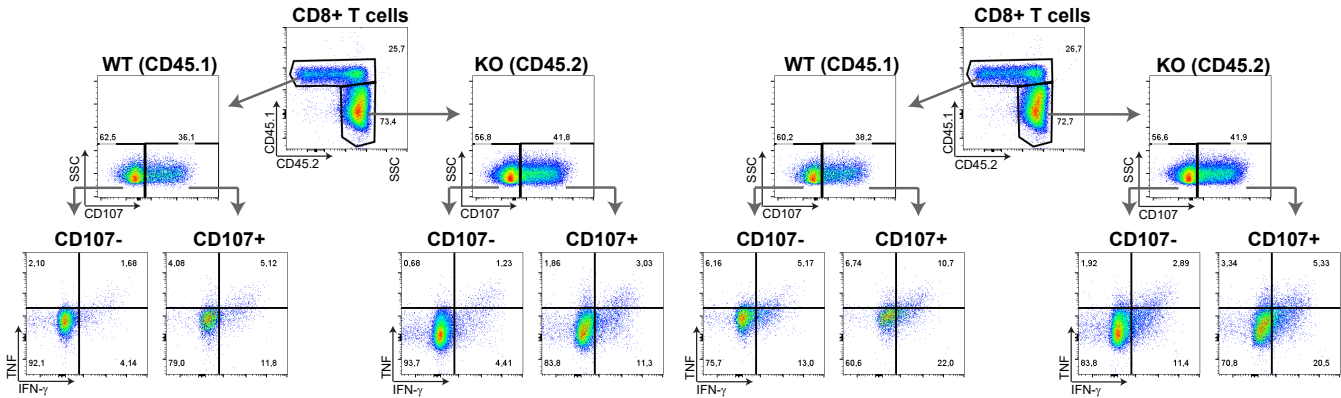

Supplement: Supplementary Figure 1 — Complementary evaluation of parasite-specific CD8+ T cell responses and tissue parasitism in T. cruzi-infected IL-17A/IL-17F DKO mice. (A) Relative amount of T. cruzi satellite DNA in spleen and liver of infected WT and IL-17A/IL-17F DKO mice determined at 22 dpi. Murine GAPDH was used for normalization. (B) Representative plots and statistical analysis of CD8 and TSKB20/Kb staining in spleen of WT and IL-17A/IL-17F DKO mice at 22 dpi. Numbers on plots represent the frequency of TSKB20/Kb+ CD8+ T cells. (C–D) Representative histograms and statistical analysis of the geometric mean expression of the inhibitory receptor PD-1 (C) and the death receptor CD95/Fas (D) in total and TSKB20/Kb+ spleen CD8+ T cells from WT and IL-17A/IL-17F DKO mice at 22 dpi. Gray tinted histogram show staining in CD8+ T cells from non-infected WT mice. Data in statistical analysis (A–D) are presented as mean ± SD, N = 4−6 mice. P values calculated with two-tailed T test. (A–D) Data are representative of at least three independent experiments. [file Data_Sheet_1.PDF]
